# Accurate quantification of spliced and unspliced transcripts for single-cell RNA sequencing with tidesurf

**DOI:** 10.1371/journal.pone.0355867

**Published:** 2026-08-17

**Authors:** Jan T. Schleicher, Doreen Klingler, Manfred Claassen

**Affiliations:** 1 Department of Internal Medicine I, University Hospital Tübingen, Tübingen, Germany; 2 M3 Research Center, University Hospital Tübingen, Tübingen, Germany; 3 Department of Computer Science, University of Tübingen, Tübingen, Germany; 4 Institute for Bioinformatics and Medical Informatics, University of Tübingen, Tübingen, Germany; Universitatsklinikum Hamburg-Eppendorf, GERMANY

## Abstract

RNA velocity enables predicting future cellular states from single-cell RNA-sequencing (scRNA-seq) data by inferring the derivative of gene expression from separately quantified spliced and unspliced transcripts. Although the original implementation, velocyto, established the foundation for such analyses and remains widely used, it has not been updated to accommodate major advances in scRNA-seq technologies. Notably, some modern 10x Genomics protocols capture transcripts from the 5’ end rather than the 3’ end, introducing reversed transcript orientations and other protocol-specific features not implemented in velocyto. We demonstrate that velocyto systematically assumes opposite transcript direction compared to 10x Genomics Cell Ranger in 5’-sequencing data, leading to different count assignments than for other tools, substantial deviations in inferred velocities, and ultimately divergent biological interpretations. To address these shortcomings, we present tidesurf, a command-line tool designed for accurate quantification of spliced and unspliced molecules across both 3’- and 5’-based scRNA-seq protocols. By evaluating tidesurf on four publicly available 10x Genomics Chromium datasets and comparing it to state-of-the-art quantification approaches, we show that it reliably recovers correct transcript counts in settings where velocyto performs well (3’ chemistry) and where it produces unexpected results (5’ chemistry). These results underscore that the validity of RNA velocity analyses critically depends on reliable splicing-state quantification and that continued use of velocyto with 5’-sequencing data is inadvisable. Tidesurf provides a robust, up-to-date alternative that preserves the interpretability and reliability of RNA velocity across diverse experimental designs.

## Introduction

Over the last decade, single-cell RNA sequencing (scRNA-seq) has established itself as a method routinely used to study dynamic processes of cells in development, differentiation, diseases, and immune responses [1,2].

The first droplet-based scRNA-seq protocols were based on capturing the poly-A tail at the 3’ end of transcripts through oligo-dT nucleotide-coated gel beads. More recently, additional protocols have been developed to capture the 5’ end of mRNA molecules, enabling, for example, the concurrent analysis of T or B cell receptor repertoires. In both cases, in the following denoted 5’ and 3’ scRNA-seq, sequencing of the gene expression library results in two separate read files, with the first read containing the cell barcode (CBC) and unique molecular identifier (UMI) and the second read containing the captured mRNA sequence. The resulting data is used to generate a cell-by-gene read count matrix. This requires read alignment to a reference genome and transcript quantification. The scRNA-seq protocols are implemented on the 10x Genomics Chromium platform, which further provides the Cell Ranger software based on the STAR aligner to achieve the alignment and quantification tasks [3,4]. While Cell Ranger considers both exonic and intronic read alignments, it does not quantify them separately but instead produces a single gene expression matrix.

La Manno *et al.* first described how a separate quantification of those reads can be leveraged for the analysis of dynamic cellular processes from snapshot data [5]. Their tool, velocyto, produces distinct count matrices for spliced, unspliced, and ambiguously assigned mRNA molecules, based on the Cell Ranger Binary Alignment Map (BAM) files. Other tools, such as alevin-fry [6] and kallisto-bustools [7], based on the pseudo-aligners salmon [8] and kallisto [9], respectively, are also capable of producing separate count matrices, using the raw sequencing FASTQ files as input. From these matrices, so-called RNA velocity, the temporal derivative of gene expression, can be estimated through a simple differential equation model [5].

While the estimation of RNA velocity has been refined by many other methods such as scVelo [10], it is usually still based on spliced and unspliced counts computed with velocyto. This is potentially problematic as velocyto has not been updated since the beginning of 2019, despite the significant progress in scRNA-seq protocols. For example, it does not offer a mode for the analysis of 10x Genomics 5’ scRNA-seq data, resulting in unexpected quantification results when used on such datasets. Nonetheless, a considerable number of studies applied velocyto to 5’ scRNA-seq data [11–18].

In this study, we re-analyzed two publicly available scRNA-seq datasets, generated with 5’ protocols and originally processed by Cell Ranger and velocyto [11,13], to assess whether velocyto could accurately quantify spliced and unspliced reads for 5’ data. We show that, compared to Cell Ranger, velocyto severely underestimates the total number of transcripts and assigns gene counts to overlapping genes on the opposite strand, assuming a different orientation of sequencing reads. To solve this issue, we present a **T**ool for **ID**entification and **E**numeration of **S**pliced and **U**nspliced **R**ead **F**ragments (tidesurf) from the Cell Ranger BAM files, which, unlike velocyto, was developed for both 3’ and 5’ data generated with 10x Genomics Chromium. We applied tidesurf to the aforementioned datasets, as well as to two further datasets based on the 3’ protocol [19,20], to demonstrate its utility. Comparing the results to the analysis of the same datasets with velocyto, alevin-fry, and STARsolo, we demonstrated its accuracy concerning the quantification of spliced, unspliced, and ambiguous counts. Furthermore, we could show that RNA velocities estimated from spliced and unspliced counts computed with tidesurf were in line with previous results. In contrast, the velocity estimates based on velocyto’s quantification for 5’ data differed considerably from those estimated from the output of the other tools, leading to different biological interpretations.

## Results

### Total counts estimated by velocyto markedly deviate from Cell Ranger for 5’ scRNA-seq data

**Fig 1:**
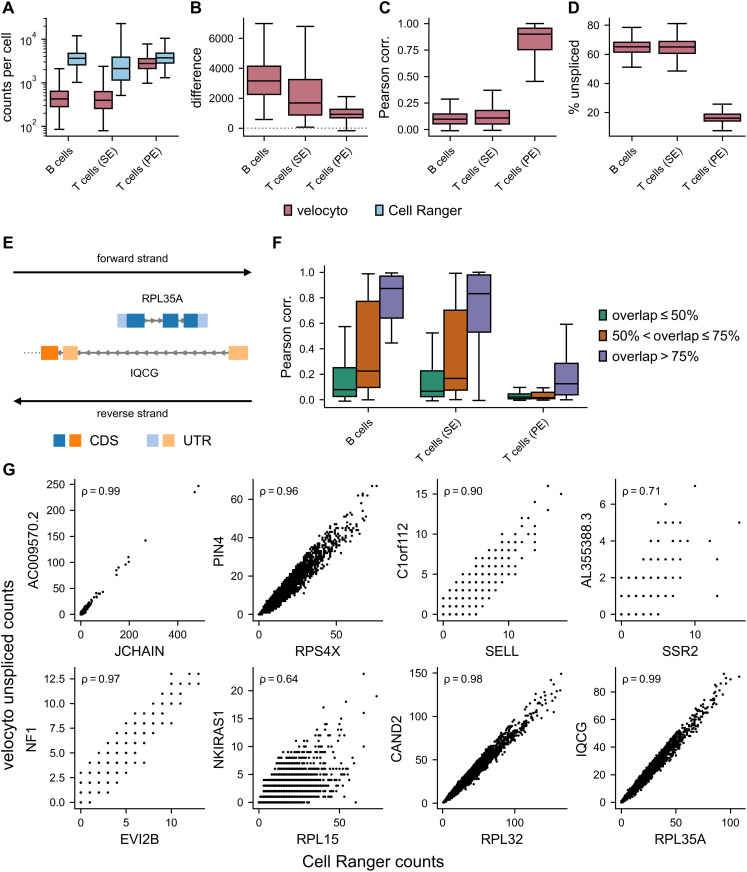
Strong disagreements between Cell Ranger and velocyto quantifications. (A-D) Boxplots of cell- or gene-wise statistics for the velocyto quantification for two 5’ scRNA-seq datasets [11,13]. The T cell dataset [13] is split into samples mapped by single-end (SE) and paired-end (PE) alignment. (A) Total counts per cell obtained from Cell Ranger and velocyto, respectively. (B) Difference of velocyto total counts per cell from the Cell Ranger total counts. (C) Pearson correlation per gene of Cell Ranger and velocyto counts. (D) Proportion of unspliced counts in the sum of spliced and unspliced counts per cell for velocyto. (E) Illustration of the overlap of the gene *RPL35A* on the forward strand with an intron of the gene *IQCG* on the reverse strand. Grey arrows represent introns and indicate the direction of the respective transcripts. Colored boxes represent exons, split into coding DNA sequence (CDS, darker shades) and untranslated regions (UTR, lighter shades). (F) Pearson correlation of Cell Ranger counts and velocyto unspliced counts of overlapping genes on opposite strands. Gene pairs are grouped by the proportion of overlapping bases, relative to the gene corresponding to the Cell Ranger counts. The T cell dataset [13] is split into samples mapped by single-end (SE) and paired-end (PE) alignment. (G) Correlation of Cell Ranger counts and velocyto unspliced counts of overlapping genes on opposite strands for the B cell dataset [11] (ρ: Pearson correlation). Genes on the x-axis are sorted in descending order of the proportion of their nucleotides overlapping with a gene located on the antiparallel strand (displayed on the y-axis).

To test the hypothesis of velocyto’s incompatibility with the analysis of 10x Genomics 5’ scRNA-seq data, we estimated spliced and unspliced counts from Cell Ranger output using velocyto for two datasets of peripheral B cells [11] and alloreactive T cells [13], generated with the 5’ chemistry. Because of differing read lengths, Cell Ranger used paired-end (PE) alignment for some samples of the T cell dataset with longer reads, while only R2 was used for alignment of the other samples (single-end, SE). We compared the sum of spliced, unspliced, and ambiguous counts of each cell, obtained by velocyto, to the total Cell Ranger counts (Fig 1A), splitting the T cell data into SE and PE samples. Velocyto yielded considerably lower counts per cell, especially for the R2-only aligned samples of both datasets, where the total count distributions differed by an order of magnitude. This was even more apparent in the per-cell differences in total counts obtained from velocyto, compared to Cell Ranger (Fig 1B). For the PE samples of the T cell dataset, there were also marked differences, albeit lower than those seen for the SE samples.

Furthermore, the median Pearson correlation of Cell Ranger counts of a gene with its velocyto total counts was around 0.1 for the SE samples of the B cell and T cell datasets (Fig 1C). For the PE samples of the T cell dataset, the correlation was considerably higher, with a median of 0.9.

Additionally, we observed unusually high proportions of unspliced counts (Fig 1D). For the B cell dataset and the SE samples of the T cell dataset, the median unspliced proportions were above 60 %. For the PE samples of the T cell data, the proportion of unspliced counts was in the range of 15–25 % reported by La Manno *et al. [5]*, with a median below 20 %.

Taken together, these results demonstrate that velocyto produces unexpected results on 10x Genomics 5’ scRNA-seq data, particularly for single-end aligned samples.

### Velocyto assigns unspliced counts to overlapping genes

Next, we investigated the mechanistic causes of these discrepancies. While velocyto assumes strand-specific reads, it does not take into account that the orientation of reads differs between 5’ and 3’ protocols. We hypothesized that the observed unspliced counts do not arise from the genes to which Cell Ranger assigns the reads, but rather from transcripts on the opposing DNA strand at the same position. This phenomenon is common in the human and mouse genomes [21]. The majority of overlaps occur between intronic regions of one gene and exons of another gene, exemplified by the human gene *RPL35A* on the forward strand, which is fully embedded within the first intron of the gene *IQCG* on the reverse strand (Fig 1E). Indeed, we observed a high correlation of unspliced gene counts assigned by velocyto with the Cell Ranger counts of the respective overlapping gene (Fig 1F–1G and S1). The degree of correlation is related to the overlap length, relative to the gene corresponding to the Cell Ranger counts (Fig 1F). Moreover, the correlation did not originate from co-expression of the genes (S2 Fig). The correlation was considerably lower for the PE samples of the T cell dataset (Fig 1F). The human and mouse genomes also contain gene pairs on opposite strands exhibiting exon-exon overlaps, at considerably lower frequency than exon-intron overlaps [21]. For example, the exons of the human gene *MIF* overlap with the last exon of the long non-coding RNA *MIF-AS1* (S1B Fig). In this case, velocyto assigned counts to the spliced transcript of the overlapping gene (S1C and S1D Fig).

In conclusion, velocyto assumed strand-specific reads for 10x Genomics scRNA-seq data, but misinterpreted the read direction relative to the transcript annotations. As a result, it frequently did not assign reads to the same gene as Cell Ranger, but to another, overlapping gene on the opposite strand.

### Tidesurf, a tool for splice state-aware quantification

**Fig 2:**
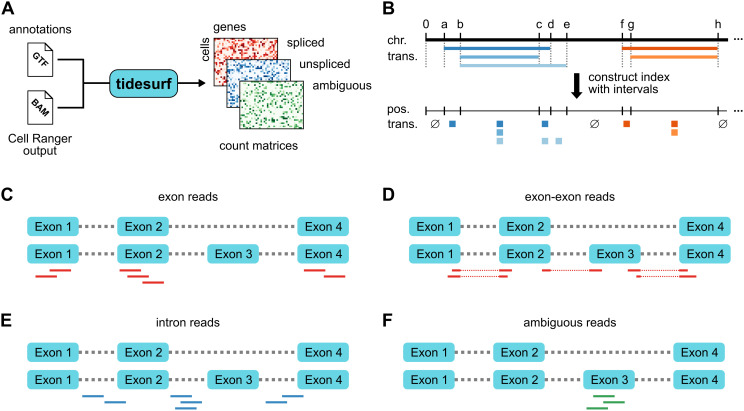
Overview of the tidesurf algorithm. (A) Tidesurf takes a GTF file with transcript annotations and the Cell Ranger output, containing the alignments in BAM format, as input and produces count matrices for spliced, unspliced, and ambiguous mRNA molecules. (B) The annotated genome is used to construct an index. To this end, a list of intervals represented by their genomic starting position is constructed, where for each interval, the set of overlapping transcripts is stored. (C-F) Examples of reads for the different read types used in tidesurf. Two example transcripts with different final exon compositions are shown. Exon reads overlap with exons for all transcripts for a gene (C), while exon-exon reads span junctions between exons (D). Intron reads are those aligned to introns for all transcripts (E), while ambiguous reads map to exons for some of the transcripts, and to introns for others (F).

To address the above quantification issues, we propose a **T**ool for **ID**entification and **E**numeration of **S**pliced and **U**nspliced **R**ead **F**ragments (tidesurf). Similar to velocyto, tidesurf uses gene annotations in General Transfer Format (GTF) and Cell Ranger-aligned reads in Binary Alignment Map (BAM) format to separately quantify spliced, unspliced, and ambiguous transcripts (Fig 2A). To efficiently retrieve transcripts overlapping with aligned sequencing reads, we use a transcript index. The index consists of a list of genomic intervals represented by their genomic starting position and the set of transcripts overlapping each interval (Fig 2B).

After retrieving transcripts, we determine the splice state by evaluating whether reads map to exonic or intronic regions, accounting for splice isoforms (Fig 2C-F). Our algorithm distinguishes between four mapped read types. (1) Exon reads map exclusively to exonic regions for all transcript isoforms, (2) exon-exon reads align to both flanks of an intron, spanning an exon-exon-junction, (3) intron reads overlap intronic regions for all isoforms, and (4) ambiguous reads align to exonic regions for a subset of transcripts, and to intronic regions for the others. The splice state of the transcript is determined based on the mapping behavior of its corresponding read types. For method details, refer to Implementation of tidesurf.

### Counts obtained with tidesurf recapitulate the Cell Ranger quantification

To demonstrate the utility of tidesurf, we analyzed four publicly available datasets generated with the 10x Genomics Chromium technology.

The pancreatic endocrinogenesis [19] and the developing retina datasets [20] were generated with the 3’ chemistry, while the peripheral B cell [11] and alloreactive T cell datasets [13] were generated with the 5’ chemistry. For all four datasets, we compared the read count output of tidesurf to the Cell Ranger expression matrix and quantifications obtained with the tools velocyto [5], alevin-fry [6], and STARsolo [4]. Further, we compared the RNA velocity estimates based on the quantifications by the competing approaches.

**Fig 3:**
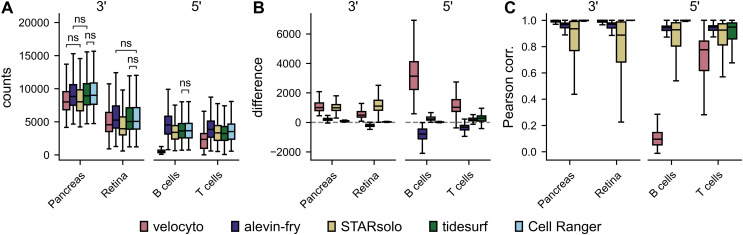
Comparison of total counts across methods. (A) Boxplot of total counts per cell (sum of spliced, unspliced, and ambiguous for velocyto, alevin-fry, and tidesurf) per dataset. Pairwise Mann-Whitney *U* test with Bonferroni multiple testing correction for *m* = 40 tests. Non-significant comparisons are marked with “ns”. All other tests are significant at α=0.05. (B) Boxplot of differences of total counts per cell from Cell Ranger counts. (C) Boxplot of per-gene Pearson correlation of counts with Cell Ranger counts.

First, we assessed the total read count outputs. For the 3’ datasets, the distribution of total counts per cell (sum of spliced, unspliced, and ambiguous counts for velocyto) was very similar between all four methods and Cell Ranger, with velocyto and STARsolo showing slightly lower total counts (Fig 3A). In contrast, velocyto total counts were considerably lower than those of alevin-fry, tidesurf, STARsolo, and Cell Ranger for the 5’ datasets, especially for the B cell data [11]. Due to the large sample size (high number of cells), most total count distributions were significantly different from each other between the different methods (pairwise two-sided Mann-Whitney *U* test with Bonferroni multiple testing correction, α=0.05, Table 1). The same trend was observed for the difference of total counts per cell of velocyto, alevin-fry, STARsolo, and tidesurf from Cell Ranger (Fig 3B). All differences were significantly different from 0, regardless of the magnitude (one-sample Student’s *t*-test with Bonferroni mul*t*iple testing correction, α=0.001). The median difference between tidesurf total counts and Cell Ranger was less than 100 for all datasets except for the T cell data, where it was slightly higher at around 280. For alevin-fry, the absolute median difference ranged between approximately 200 on the 3’ datasets and almost 800 on the B cell data. For the STARsolo quantification, the median difference was around 200 for the two 5’ datasets, but around 1,000 for the 3’ datasets. Velocyto’s median difference was around 500 for the retina data, around 1,000 for the pancreas and T cell datasets, and above 3,000 for the B cell data. The counts showed a high Pearson correlation with the Cell Ranger counts of the same gene for alevin-fry, STARsolo, and tidesurf on all four datasets (median correlation always above 0.88), while for velocyto, this was only the case for the 3’ datasets (Fig 3C).

In summary, the sum of tidesurf’s spliced and unspliced counts closely resembled the Cell Ranger counts on all evaluated datasets. In contrast, alevin-fry and STARsolo showed slightly larger discrepancies, and velocyto strongly deviated from Cell Ranger for the two 5’ datasets.

**Table 1:** Test results for the comparison of total count distributions across methods. Pairwise Mann-Whitney *U* test with Bonferroni multiple testing correction for *m* = 40 tests. *: *p* < 0.05, **: *p* < 0.01, ***: *p* < 0.001, ns: not significant at α=0.05. Method name abbreviations in the columns: V: velocyto, A: alevin-fry, S: STARsolo, T: tidesurf.

Dataset	Pancreas				Retina				B cells				T cells			
Method	V	A	S	T	V	A	S	T	V	A	S	T	V	A	S	T
alevin-fry	***				***				***				***			
STARsolo	ns	***			***	***			***	***			***	***		
tidesurf	***	ns	***		***	*	***		***	***	***		***	***	***	
Cell Ranger	***	***	***	ns	***	ns	***	ns	***	***	***	ns	***	***	***	***

### Spliced, unspliced, and ambiguous counts are similar between tidesurf, alevin-fry, and STARsolo

**Fig 4:**
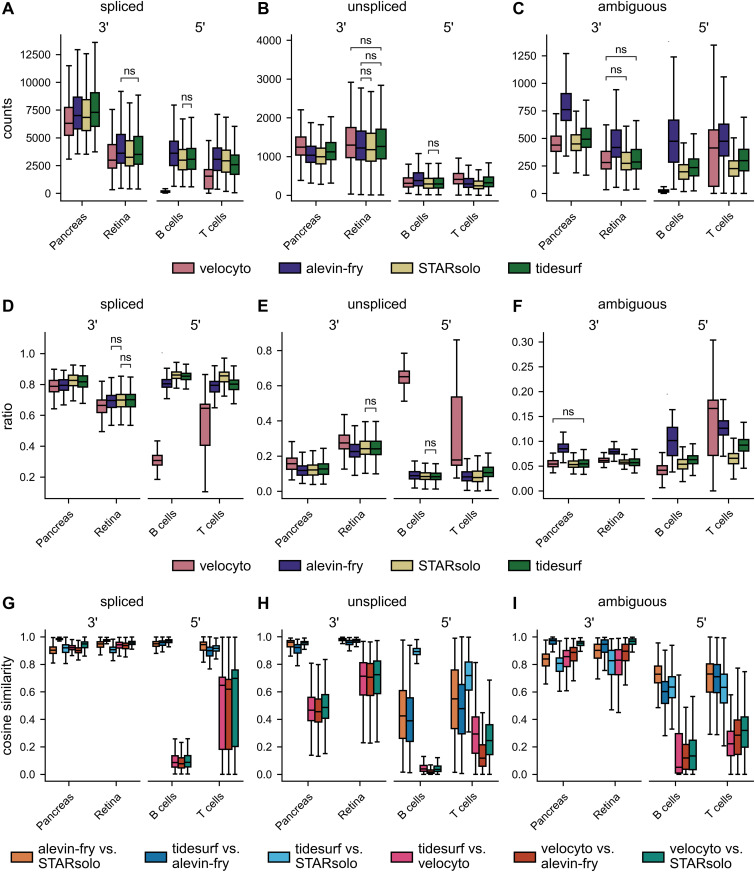
Comparison of spliced, unspliced, and ambiguous counts across methods. (A-C) Boxplots of total spliced (A), unspliced (B), and ambiguous (C) counts per cell. (D-F) Boxplots of ratios of spliced (D), unspliced (E), and ambiguous (F) counts out of total counts per cell. Panels A-F: Pairwise Mann-Whitney *U* test with Bonferroni multiple testing correction for *m* = 24 tests. Non-significant comparisons are marked with “ns”. All other tests are significant at α=0.05. (G-I) Boxplots of cosine similarity of spliced (G), unspliced (H), and ambiguous (I) counts per cell between methods.

Furthermore, we separately compared the total spliced, unspliced, and ambiguous counts per cell before normalization. For the 3’ datasets, the distributions of total spliced counts were similar between all four methods (Fig 4A). In contrast, velocyto produced considerably lower spliced counts than alevin-fry, STARsolo, and tidesurf for the 5’ datasets. The distributions of unspliced counts were comparable between all tools on all four datasets, regardless of the assay chemistry (Fig 4B). Alevin-fry tended to report higher ambiguous counts than velocyto, STARsolo and tidesurf (Fig 4C). Due to the large sample size (high number of cells), most spliced, unspliced, and ambiguous count distributions were significantly different from each other between the different methods (pairwise two-sided Mann-Whitney *U* test with Bonferroni multiple testing correction, α=0.05, Table 2). Accordingly, the total counts differences per cell from the tidesurf quantification were low for spliced counts for alevin-fry and STARsolo on all four datasets, but more pronounced for velocyto on the 5’ datasets (S3A Fig). On the pancreas and retina datasets, velocyto quantified more molecules as unspliced than tidesurf (S3B Fig) and achieved similar total ambiguous counts (S3C Fig). STARsolo yielded lower unspliced and similar ambiguous counts as tidesurf on all datasets except for the B cell data, where unspliced counts were very similar to tidesurf. In contrast, alevin-fry produced lower total spliced counts and higher ambiguous counts on all four datasets. This difference may stem from the different resolution strategies for UMI splice types. In contrast to velocyto and tidesurf, alevin-fry bases its assignment of a UMI to a splice type on a majority vote of its corresponding reads. In all cases, regardless of the magnitude, the differences from tidesurf were significantly different from 0 (one-sample Student’s *t*-test with Bonferroni mul*t*iple testing correction, *p* < 0.001 for all methods except for velocyto on the B cell data, where *p* < 0.01).

**Table 2:** Test results for the comparison of spliced, unspliced, and ambiguous count distributions across methods. Pairwise Mann-Whitney *U* test with Bonferroni multiple testing correction for *m* = 24 tests per splice state. *: *p* < 0.05, **: *p* < 0.01, ***: *p* < 0.001, ns: not significant at α=0.05. Method name abbreviations in the columns: V: velocyto, A: alevin-fry, S: STARsolo.

	Dataset	Pancreas			Retina			B cells			T cells		
Splice state	Method	V	A	S	V	A	S	V	A	S	V	A	S
spliced	alevin-fry	***			***			***			***		
	STARsolo	***	**		***	***		***	***		***	***	
	tidesurf	***	***	***	***	ns	***	***	***	ns	***	***	***
unspliced	alevin-fry	***			***			***			***		
	STARsolo	***	***		***	ns		***	***		***	***	
	tidesurf	***	***	***	ns	ns	***	***	***	ns	***	***	***
ambiguous	alevin-fry	***			***			***			***		
	STARsolo	**	***		ns	***		***	***		***	***	
	tidesurf	***	***	***	ns	***	**	***	***	***	***	***	***

Similarly, the ratios of spliced (Fig 4D), unspliced (Fig 4E), and ambiguous counts (Fig 4F) per cell out of all counts were in a comparable range for all methods on the 3’ datasets, while velocyto had much lower proportions of spliced and much higher proportions of unspliced molecules for the 5’ datasets. Nonetheless, as for the count distribution, most of the ratio distributions were significantly different between the different methods (pairwise two-sided Mann-Whitney *U* test with Bonferroni multiple testing correction, α=0.05, Table 3).

**Table 3:** Test results for the comparison of spliced, unspliced, and ambiguous ratio distributions across methods. Pairwise Mann-Whitney *U* test with Bonferroni multiple testing correction for *m* = 24 tests per splice state. *: *p* < 0.05, **: *p* < 0.01, ***: *p* < 0.001, ns: not significant at $\begin{array}{c} \alpha =0.05 \end{array}$. Method name abbreviations in the columns: V: velocyto, A: alevin-fry, S: STARsolo.

	Dataset	Pancreas			Retina			B cells			T cells		
Splice state	Method	V	A	S	V	A	S	V	A	S	V	A	S
spliced	alevin-fry	***			***			***			***		
	STARsolo	***	***		***	ns		***	***		***	***	
	tidesurf	***	***	***	***	**	ns	***	***	***	***	***	***
unspliced	alevin-fry	***			***			***			***		
	STARsolo	***	*		***	***		***	***		***	***	
	tidesurf	***	***	***	***	***	ns	***	***	ns	***	***	***
ambiguous	alevin-fry	***			***			***			***		
	STARsolo	***	***		***	***		***	***		***	***	
	tidesurf	ns	***	***	***	***	***	***	***	***	***	***	***

For a direct, pairwise comparison between methods, we investigated the per-cell cosine similarities and per-gene Pearson correlations of spliced, unspliced, and ambiguous counts, respectively. For this analysis, we only considered genes present for all three methods. On the 3’ datasets, the median cosine similarity of spliced counts between tidesurf and alevin-fry was close to 1, and above 0.9 for all other pairs of methods (Fig 4G). In contrast, the median similarity between velocyto and any other method was below 0.1 for the B cell data and between 0.6 and 0.7 for the T cell data, but above 0.89 for all pairwise comparisons not involving velocyto. The median Pearson correlation was above 0.95 for all pairwise comparisons on the pancreas and retina datasets, and between 0.89 and 0.98 for the pairwise comparisons of all methods except for velocyto on the B cell and T cell datasets (S3D Fig). Like the cosine similarity, the Pearson correlation between velocyto’s spliced counts and those of the other two methods was also considerably lower on the 5’ datasets, especially on the B cell data. For unspliced counts, the median pairwise cosine similarities between tidesurf, alevin-fry, and STARsolo were above 0.92 on the 3’ datasets. For the 5’ datasets, the median similarities were around 0.4 and 0.5 for comparisons of alevin-fry and STARsolo or tidesurf, and around 0.9 and 0.7 between tidesurf and STARsolo on the B cell and T cell data, respectively. Velocyto had a lower similarity to the other three methods for all datasets, with particularly low values for the B cell data (median below 0.05, Fig 4H). A similar trend was observed for gene-wise Pearson correlations on the 5’ datasets, while velocyto had slightly higher correlations to tidesurf than the other comparisons on the 3’ data (S3E Fig). Ambiguous counts had a median cosine similarity above 0.8 on the pancreas and retina datasets for all comparisons, median cosine similarities between 0.6 and 0.75 on the 5’ datasets for all comparisons not involving velocyto (Fig 4I). The correlations of the ambiguous counts showed a similar trend (S3F Fig). The similarity and correlation between velocyto and the other three methods were considerably lower. As described above, the considerable differences between velocyto and the other three tools on the 5’ datasets were expected since velocyto’s quantification also differed considerably from Cell Ranger for these protocols (Figs 1 and S1).

There are multiple plausible reasons for the higher differences between alevin-fry and tidesurf on the 5’ datasets compared to the 3’ datasets. First, the 3’ datasets stem from murine samples, while the 5’ datasets originate from human donors. The human genome contains more repetitive sequences than the murine genome [22], which could lead to differences in the (pseudo-)alignments of Cell Ranger and alevin-fry, especially for intronic sequences. Furthermore, the total unspliced counts were considerably lower on the two 5’ datasets, rendering small absolute differences relatively more important. Finally, alevin-fry’s quantification of total counts differed slightly more from the Cell Ranger output than tidesurf’s (Fig 3B–3D).

### RNA velocities and their interpretation are similar between quantification methods except for velocyto on 5’ data

Finally, we computed RNA velocities based on the spliced and unspliced counts obtained from the four different tools. To reduce the impact of other factors, we used the same PCA embedding and neighborhood graph for each method. For visualization, the velocities were projected onto the UMAP embedding obtained from the original processed dataset of the respective reference publication. To highlight differences between the methods, we computed the cosine similarity of the projected, two-dimensional velocities between tidesurf and the other three methods for each cell.

**Fig 5:**
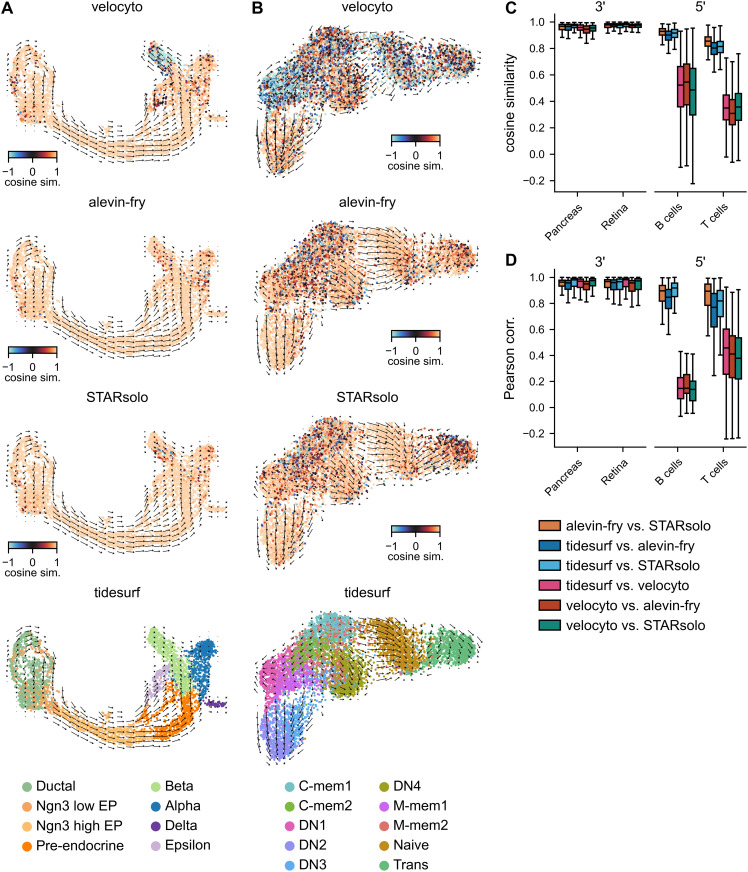
Comparison of RNA velocities between methods. (A-B) Visualization of velocities for the pancreatic endocrinogenesis [19] (A) and B cell [11] (B) datasets, computed from spliced and unspliced counts obtained with velocyto, alevin-fry, STARsolo, or tidesurf (top to bottom) on a two-dimensional UMAP embedding. In the top three subpanels, color represents the cosine similarity between two-dimensional UMAP velocity projections between the respective method and tidesurf. In the bottom panel, color represents cell type. (C) Boxplot of cosine similarity of velocity vectors per cell between methods for velocity genes. (D) Boxplot of Pearson correlation of velocities per gene between methods for velocity genes.

For the pancreatic endocrinogenesis dataset, the velocity embedding is very similar between all four methods (Fig 5A). In particular, there is always a cyclical component in the ductal cells on the left side of the embedding and a unidirectional flow through the endocrine progenitor (EP) and pre-endocrine cells at the bottom. The cosine similarity to the projected velocities based on tidesurf’s quantification is high for all three methods for almost all cells. The only major difference lies in the β-cells, where the grid arrows in the velocyto embedding all point towards the top left corner of the cluster, coinciding with a negative cosine similarity to tidesurf in this region, while the arrows in the top left part of the cluster point towards the lower right for alevin-fry, STARsolo, and tidesurf. We speculate that this localized discrepancy might arise from small upstream differences in the ratio of spliced and unspliced counts, in particular for β-cell-related genes, as well as from the selection of different velocity genes. Especially in differentiated cells close to the steady state, such small variations in a handful of cell-type-defining genes could lead to reversed projected velocities.

On the visualization of the developing retina dataset, there are no discernible differences, with high cosine similarity across the embedding (S4A Fig). In contrast, the embedded velocities based on velocyto differ more strongly from those based on alevin-fry, STARsolo, and tidesurf on the UMAPs of the 5’ datasets. For example, they are different in the DN1, DN4, and Naive clusters of the B cell data (Fig 5B). Across the embedding of the velocyto-based velocities, a large proportion of the cells show a negative cosine similarity to tidesurf. While there are also cells with low similarity for alevin-fry and STARsolo, their number is much lower, and the majority of cells have a high similarity. Similarly, there were many cells with velocities pointing in the opposite direction for velocyto compared to tidesurf on the T cell data (S4B Fig). The differences between alevin-fry or STARsolo and tidesurf were less pronounced.

Additionally, we directly compared cell-wise, high-dimensional velocity vectors. For this analysis, we focused on so-called “velocity genes”, that is, genes with a sufficiently good fit of scVelo’s model, which are used for downstream analyses, including the computation of a transition probability matrix and the two-dimensional representation of velocities. Since the sets of velocity genes differed across quantification methods, we used the pairwise union of velocity genes for each method, excluding genes filtered out in either quantification due to low counts. The number of genes used in the respective pairwise comparisons, as well as the number of velocity genes per quantification, are shown in Table 4. All pairwise cosine similarities were above 0.94 in the median on the pancreas and retina datasets (Fig 5C). For the 5’ datasets, cosine similarities were lower. Here, the highest similarities were observed in the comparisons between tidesurf, alevin-fry, and STARsolo (median above 0.9 and above 0.8 for B cells and T cells, respectively), whereas the velocities obtained based on the velocyto quantification were considerably less similar. The median gene-wise Pearson correlation out of the pairwise union of velocity genes was above 0.95 for all comparisons on the 3’ datasets, and above 0.76 between tidesurf, alevin-fry, and STARsolo on the 5’ data (Fig 5D). Velocities based on velocyto had considerably lower correlations to those from the other tools.

**Table 4:** Number of velocity genes in the analyzed datasets. For each dataset, the diagonal shows the number of velocity genes based on the respective quantification. The below-diagonal elements indicate the number of genes in the pairwise union of velocity genes between the respective tools, excluding those filtered out for one of the compared quantifications because of low spliced and unspliced counts. Method name abbreviations in the columns: V: velocyto, A: alevin-fry, S: STARsolo, T: tidesurf.

Dataset	Pancreas				Retina				B cells				T cells			
Method	V	A	S	T	V	A	S	T	V	A	S	T	V	A	S	T
velocyto	1,177				1,042				10				266			
alevin-fry	1,189	1,102			1,072	1,001			36	162			492	443		
STARsolo	1,207	1,166	1,009		1,089	1,069	852		32	183	153		510	525	472	
tidesurf	1,214	1,180	1,191	1,167	1,080	1,072	1,099	1,049	37	192	187	182	547	573	590	532

Overall, RNA velocities based on the different tools were very similar for the 3’ datasets, both in gene expression space and on the two-dimensional projection. For the 5’ datasets, the differences between alevin-fry, STARsolo, and tidesurf were more pronounced, but the RNA velocities still showed high similarities. In contrast, the velocyto-derived velocities differed considerably from those derived from any of the other tools.

When comparing velocity vectors for all pairwise common genes, observed cosine similarities were considerably lower, especially on the 5’ datasets (S4C Fig). Only the similarity between STARsolo and tidesurf was at a high level for the B cell and T cell data, with a median of approximately 0.9 and 0.8, respectively. Gene-wise Pearson correlations showed similar distributions for all genes as for velocity genes (S4D Fig). We assume that the drop in cosine similarity is related to the well-known phenomenon that cosine similarities become more sensitive to small differences with increasing dimensionality [23]. Further, we presume that the higher differences between velocities based on alevin-fry, STARsolo, and tidesurf, respectively, on the 5’ datasets can be attributed to the differences in unspliced counts described above, as they significantly impact velocity estimation. Additionally, the B cell and T cell datasets might be less suitable for RNA velocity analysis than the pancreas and retina datasets. While the latter have been used in many RNA velocity analyses, showing biologically meaningful results, we selected the former because they had been analyzed with velocyto by the original authors, yielding possibly erroneous velocity estimates. A strong indication of the unsuitability of these datasets for RNA velocity is their generally lower number of velocity genes (below 200 and around 500 for the B and T cell data, respectively, and approximately 1,000 for the 3’ datasets, see Table 4), showing that fewer genes had a good fit for scVelo’s velocity model.

### Cluster transitions inferred by PAGA differ depending on the quantification method

**Fig 6:**
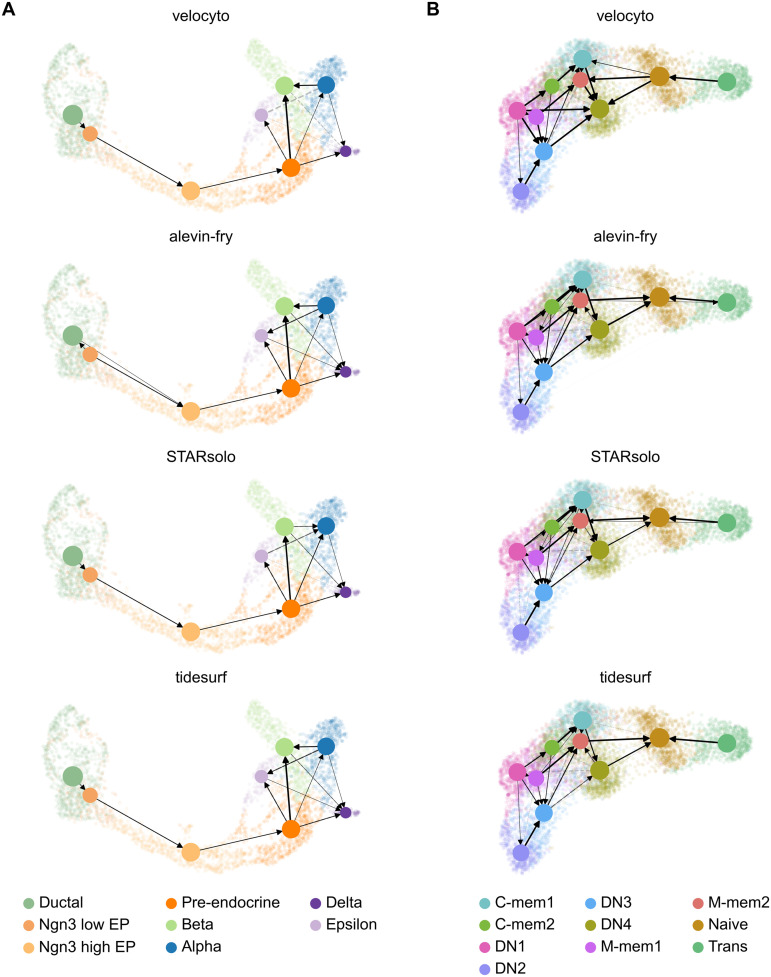
Comparison of inferred cluster transitions. (A-B) Partition-based graph abstraction (PAGA) [24] with velocity-directed edges for the pancreatic endocrinogenesis [19] (A) and B cell [11] (B) datasets. The underlying RNA velocity estimates were computed from the spliced and unspliced counts obtained with velocyto, alevin-fry, STARsolo, and tidesurf, respectively (top to bottom). Dashed lines show connectivities, and solid arrows indicate transitions.

As an example for the influence of differing RNA velocity estimates on downstream trajectory inference tasks, we performed partition-based graph abstraction (PAGA) [24] with velocity-directed edges. For the pancreas dataset, the general PAGA-inferred cluster transition trend was the same for all four methods, highlighting the transition from Ngn3 low EP cells to the four differentiated endocrine cell types (alpha, beta, epsilon, and delta) via Ngn3 high EP and pre-endocrine cells (Fig 6A). However, there were some differences in the inferred transitions between the differentiated endocrine cell types between the different methods. Additionally, PAGA inferred a transition from ductal to Ngn3 low EP cells for the velocyto-, and STARsolo-, and tidesurf-based velocities, while it detected the opposite direction for alevin-fry. This effect could be attributed to the cycling nature of the ductal cells. Similarly, PAGA inferred the same transitions from neuroblasts to all other cell types for the retina dataset (S5 Fig).

For the B cell data, the inferred PAGA transitions showed a high agreement between alevin-fry, STARsolo, and tidesurf (Fig 6B). However, the direction of the transitions between naive and M-Mem2 or DN4 was inverted in the graph inferred from velocyto-based velocities. Here, there was also an additional suggested transition from DN1 to DN4 that was not inferred for any of the other methods.

For cluster transition inference on the T cell data, we restricted the analysis to five clusters chosen by the original authors for PAGA analysis [13]. In contrast to their approach, we did not perform separate analyses for different sample types. Only a couple of transitions were inferred for all four quantification methods: from c03 to c01 and from c04 to c02 (S5B Fig). Apart from these similarities, the PAGA graphs differed considerably. Notable differences for the velocyto-based graph were the prediction of transitions from c04 to c03 and c07, which were not present for any of the other tools, as well as the transition from c02 to c01, which was inverted compared to all other methods.

In summary, trajectory inference with PAGA demonstrated the overall high concordance across quantification tools for the 3’ datasets. Furthermore, it underscored the differences for the 5’ datasets, in particular for the results based on velocyto. We assume that differences in spliced and unspliced counts propagate and accumulate through velocity estimation downstream to trajectory inference. Hence, the larger differences between the PAGA graphs on the 5’ datasets, which, as argued above, might be less suitable for RNA velocity analysis in general, are not surprising. Moreover, PAGA seems to be sensitive to even small differences in the input.

### Run time and memory requirements of tidesurf are comparable to velocyto

Lastly, we also compared the run times and memory requirements of tidesurf, velocyto, alevin-fry, and STARsolo on a cluster with AMD EPYC 7343 CPUs. We allocated 32 cores for each process. For velocyto, the threads and memory per thread for sorting with samtools were set to 32 threads and 4 GB, respectively. To obtain accurate estimates, we selected five of the 19 samples analyzed in this study, representing the quartiles of BAM file size based on the number of reads. We executed all tools five times per sample. Tidesurf had similar run times and memory requirements as velocyto (S6 Fig). In contrast, alevin-fry and STARsolo had considerably lower execution times and memory requirements. The lower run times of these tools can be attributed to the pseudo-alignment in the case of alevin-fry and the implementation in Rust (alevin-fry) and C/C++ (STARsolo).

## Discussion

The standard tool for quantification of spliced and unspliced molecules for scRNA-seq data, velocyto, was developed before 10x Genomics introduced 5’ gene expression protocols. Despite this technological progress, velocyto has not been updated, and on the velocyto GitHub repository (https://github.com/velocyto-team/velocyto.py, accessed 16.06.2026), the authors state that it is no longer maintained and should not be used anymore. Nevertheless, it was used for spliced/unspliced quantification in a plethora of studies using 5’ scRNAseq [11–18]. By reanalyzing the data from two such studies, we demonstrated that velocyto assigned reads to overlapping genes on the opposite strand compared to Cell Ranger, 10x Genomics’ own software, for 5’ scRNA-seq data, resulting in considerably underestimated total counts and highly inflated unspliced proportions. For samples where Cell Ranger aligned the reads in paired-end mode, these issues were alleviated. However, for some genes, the assignment remained inaccurate, indicating that velocyto should, in general, not be used for 10x Genomics 5’ datasets.

Therefore, we introduced tidesurf as a novel command-line tool for quantifying spliced and unspliced transcripts from 10x Genomics Chromium scRNA-seq experiments. We showed its application to four different datasets covering both 3’ and 5’ sequencing technology and demonstrated its accuracy. The accurate quantification of spliced and unspliced counts is a prerequisite for sensible RNA velocity estimates. Consequently, tidesurf represents a replacement for velocyto on 5’ data, where the latter produces count matrices that differ considerably from those produced by any other tool tested here. Additionally, we propose to use tidesurf on 3’ data as well, since it generates comparable count matrices and avoids, unlike velocyto, generating an additional BAM file sorted by cells. Tidesurf’s total counts also showed considerably lower discrepancies from the Cell Ranger expression matrix than those obtained with velocyto.

In contrast to alevin-fry and STARsolo, tidesurf can be more easily incorporated into standard pipelines where 10x Genomics Cell Ranger is commonly used for gene expression quantification. Tidesurf directly uses Cell Ranger’s output and produces spliced, unspliced, and ambiguous counts for the same cell barcodes (or a superset if filtering is turned off) in convenient AnnData format for easy integration with Cell Ranger’s expression matrix. For alevin-fry, on the other hand, it is necessary to run a second analysis from scratch, which can lead to the calling of slightly different cell barcodes. Similarly, STARsolo performs its own cell filtering steps, which may result in different cells than the Cell Ranger pipeline. While the STARsolo documentation, a single markdown document that is several versions behind the most recent release, describes parameter settings for obtaining similar results as with Cell Ranger, we still observed greater differences from Cell Ranger than when using tidesurf. Furthermore, the use of alevin-fry on Chromium 5’ data requires the adjustment of two parameters relating to read orientation with respect to each other and the reference genome in two different functions, which we only detected through a thorough search of multiple issues on the respective project repositories. In the same way, we had to make multiple adjustments for STARsolo on the 5’ data. Even with all adjustments made according to the documentation, when processing some samples with longer reads in paired-end alignment mode, STARsolo crashed with a segmentation fault, necessitating falling back to single-end alignment mode. This issue has been reported by many users on the STAR GitHub repository, which has almost 1,000 open issues. For tidesurf, only a single command line argument has to be changed to switch from the default sense orientation of reads to reference annotations for 3’ data to the antisense orientation for 5’ data. Moreover, the tidesurf quantification was closest to Cell Ranger across all tested datasets in terms of absolute count differences and expression profile similarity.

We compared velocity embeddings to assess the quality of tidesurf’s read count annotations as a basis for RNA velocity estimates. This comparison relied on RNA velocity estimates using scVelo’s stochastic model. We acknowledge that the resulting velocities are estimates, and that for these and their visualization shortcomings have been demonstrated [25,26]. The comparisons using cosine similarity and Pearson correlation offered a more detailed view and showed differences in counts and estimated velocities between the methods. However, the significance of these differences is hard to judge. In the absence of a ground truth, we cannot conclusively answer the question of which quantification is more correct. Nonetheless, on the 5’ datasets, we observed considerable differences between velocities based on velocyto and those based on any of the other methods, while differences between the other three tools were less pronounced. These differences were observed in comparisons of high-dimensional and projected velocities as well as in downstream trajectory inference. Because of this and because of the aforementioned assignment to transcripts on the opposite strand compared to Cell Ranger, we assume that the results of velocyto on the 5’ datasets were less reliable than the results based on the other quantification tools.

All of the tools described in this study, including tidesurf, make simplifying assumptions about splicing dynamics. Rather than explicitly modeling different transcript isoforms, molecules are categorized as either spliced or unspliced. This approach also does not account for partially spliced intermediates.

Finally, tidesurf is currently limited to 10x Genomics Chromium single-cell sequencing libraries processed with Cell Ranger. While Chromium is one of, if not the, most widely used platform for suspension-based scRNA-seq, we plan to adapt tidesurf to other single-cell protocols and alignment pipelines in future work. These adaptations would make it more widely applicable to replace velocyto.

## Conclusion

We demonstrate that velocyto, the seminal tool for quantification of spliced and unspliced transcripts from aligned scRNA-seq reads, systematically assumes a different read direction in 10x Genomics 5’ scRNA-seq data than Cell Ranger, leading to the assignment of counts to overlapping transcripts, affecting RNA velocity estimates, downstream analyses, and their biological interpretation, with results differing from those obtained with other tools. Therefore, we advise against using velocyto on scRNA-seq data generated with recent experimental protocols, such as 10x 5’ scRNA-seq. Instead, we propose to use our novel tool tidesurf. Alternatively, other methods enable splice state-aware quantification as well, such as alevin-fry [6], STARsolo [4], or kallisto-bustools [7]. Our analyses showed that tidesurf’s quantifications most closely resembled the results obtained with Cell Ranger. This highlights the utility of tidesurf to obtain quantifications of spliced and unspliced transcripts that complement a previous analysis with only minor discrepancies. If the analysis is started from scratch, alevin-fry and STARsolo are good options as well. However, their correct usage is slightly more complicated. In our experience, especially STARsolo is not very user-friendly, with insufficient documentation and almost 1,000 unresolved issues on the GitHub repository.

In conclusion, accurate quantifications of spliced and unspliced mRNA molecules are paramount for sensible RNA velocity analysis and trajectory inference. The correctness and plausibility of the count matrices should be investigated in detail before performing downstream analyses, especially when using outdated tools.

## Materials and methods

### Implementation of tidesurf

We developed tidesurf, a command-line tool for quantifying spliced and unspliced transcripts, implemented in Python. The tool takes a General Transfer Format (GTF) file with gene and transcript annotations for the reference genome used for read alignment and a Cell Ranger [3] output directory as input (Fig 2A).

In the first step, a transcript index is built from the GTF file to efficiently retrieve transcripts overlapping with aligned sequencing reads. To this end, all transcripts on a particular strand (plus or minus) and chromosome in the GTF file and their exons are inserted into a sorted list of intervals (Fig 2B). Each entry in the list consists of the start position of the respective interval and the set of transcripts contained therein. Hence, each interval start corresponds to the start of one or multiple transcripts or the base directly after one or multiple transcripts.

In the second step, the Binary Alignment Map (BAM) file produced by Cell Ranger is processed. The aligned reads are processed iteratively. Unmapped reads, those that have a mapping quality below 255 (i.e., not confidently mapped to a single locus), or reads that do not have a cell barcode (CBC) or unique molecular identifier (UMI) tag are discarded. All other reads are handled individually to determine the gene of origin and splicing status.

First, all transcript annotations overlapping the read are extracted from the transcript index using a binary search for the genomic start and end positions of the read alignment. Since the list of intervals and transcripts contained therein is sorted, this allows us to obtain all relevant transcripts. Here, we only take the strand into account that has the correct orientation relative to the read alignment. For 10x Genomics Chromium, this is the sense orientation for 3’ and the antisense orientation for 5’ protocols. Next, we determine for each overlapping transcript if the read maps to an exonic or intronic region (Fig 2C-F). This is done by iterating over the exons and introns of the transcript and summing up the respective base overlaps, taking the Concise Idiosyncratic Gapped Alignment Report (CIGAR) string of the aligned read into account. Furthermore, we keep track of the number of exons aligned with the read. For each transcript, we determine the read type based on the following criteria. If the number of bases not overlapping with exons is smaller than a threshold (default: 5), the read is classified as exonic (Fig 2C) or as spanning an exon-exon junction (Fig 2D), depending on whether the number of exons is 1 or greater than 1. On the other hand, if the number of bases overlapping with introns is greater than or equal to that threshold, the read is classified as intronic with respect to the transcript (Fig 2E). Finally, we determine the overall type of a read based on all transcripts. If all transcripts belong to the same gene, a read is classified as spanning an exon-exon junction if that annotation is present. Otherwise, it is classified as either intronic or exonic if the same type was inferred for all transcripts or ambiguous if the type differs between transcripts (Fig 2F). The rationale behind this is that a read aligning to a region that is an exon for one splice isoform, but an intron for another one cannot be unambiguously assigned unless it spans an exon-exon junction for one of the transcripts. This behavior only occurs when an intron is spliced out.

If the transcripts belong to multiple genes, the default is to discard the read. Alternatively, a read type can be assigned per gene as described above. In that case, it is weighted by the inverse of the number of genes for downstream processing.

In the third step, CBCs and UMIs are deduplicated. For each CBC/UMI/gene/read type combination, we compute its read support. After filtering out read types with very low counts and percentages, we retain only one read type for each barcode/UMI/gene combination. Here, we keep the first read type, where the sort order is intronic < exon-exon junction < ambiguous < exonic. Then, we map the read type to a splice type, where intronic maps to unspliced, ambiguous stays ambiguous, and the two exonic read types map to spliced.

Then, multi-mapped UMIs are resolved. Following Cell Ranger’s gene expression algorithm [27], we assign a CBC/UMI combination to the gene with the highest read support. Ties for maximal read support are discarded.

Finally, spliced, unspliced, and ambiguous UMIs are counted for each cell and gene, resulting in three separate count matrices which are combined into and saved as an AnnData object [28].

### Data retrieval

For the mouse pancreatic endocrinogenesis dataset at embryonic day 15.5 [19], FASTQ files were obtained from the Gene Expression Omnibus (GEO, sample accession number GSM3852755). Processed data was obtained from the scVelo (v0.3.3) pancreas dataset.

For the dataset of developing mouse retinal neurons [20], FASTQ files for batch F2 were downloaded from GEO (sample accession number GSM3466902). Processed data was downloaded from the dataset link provided by the authors of UniTVelo [29] on the UniTVelo GitHub repository.

Raw FASTQ files and processed data of human peripheral B cells [11] were obtained from ArrayExpress (accession number E-MTAB-9544).

BAM files and processed data of human alloreactive T cells [13] were accessed from GEO (series accession number GSE252994). The BAM files were converted to FASTQ using 10x Genomics bamtofastq.

### Cell Ranger

Raw reads were aligned to the respective annotated reference genome mouse mm10 (GENCODE vM23/Ensembl98) or human GRCh38 (GENCODE v32/Ensembl98), obtained from the Cell Ranger 2020-A reference packages, using 10x Genomics Cell Ranger (v7.1.0) count with automatic chemistry detection [3].

### Velocyto

To obtain spliced and unspliced counts with velocyto (v0.17.17), the Cell Ranger output directories were processed using run10x [5] with the respective annotation GTF file.

### Alevin-fry

Spliced + intronic (splici) references were generated from the Cell Ranger reference transcriptomes with pyroe (v0.9.3) make-splici according to the read length of the respective study (pancreas [19]: 151 bases, retina [20]: 100 bases, B cells [11]: 100 bases, and T cells [13]: 91 bases (samples MJ001, MJ002, MJ003, MJ016, and MJ017) or 101 bases (samples MJ005, MJ006, MJ007, MJ008, MJ009, MJ018, and MJ019)) with a flank trim length of 5 bases. Transcriptome indices were built with salmon (v1.10.2) index [8]. Reads were mapped against the respective index with salmon alevin with library type ISR for 3’ data and ISF for 5’ data. For quantification of spliced and unspliced reads with alevin-fry (v0.8.2), permit lists were generated using generate-permit-list with expected orientation fw for 3’ data and rc for 5’ data, and barcodes were corrected with collate [6]. Finally, quant was used for quantification. The results were transformed into an AnnData object using load_fry from pyroe. An overview of the parameter settings is shown in Table 5.

**Table 5:** Parameter settings for pyroe, salmon, and alevin-fry. For the pancreas, retina, and B cell datasets, the same parameters were used for all samples. For the T cell dataset, different settings were used for samples where Cell Ranger performed single-end (SE) and paired-end (PE) alignment.

Tool	Command	Parameter	Pancreas	Retina	B cells	T cells	
			all	all	all	SE	PE
pyroe	make-splici	read length	151	100	100	91	101
		flank trim length	5	5	5	5	5
salmon	alevin	library type	ISR	ISR	ISF	ISF	ISF
alevin-fry	generate-permit-list	orientation	fw	fw	rc	rc	rc

### STARsolo

To quantify spliced and unspliced transcript molecules with STARsolo (v2.7.11b), STAR genome indices were generated from Cell Ranger annotated reference genomes with the command STAR --runMode genomeGenerate --genomeDir path/to/genome/dir --genomeFastaFiles /path/to/genome.fa --sjdbGTFfile /path/to/genes.gtf --genomeSAsparseD 3 according to the documentation [4]. For the pancreas dataset, we additionally set the length of the sequence on each side of splice junctions to 149 with the argument --sjdbOverhang 149 to account for the read length of 150 bp as recommended, while using the default value of 100 for the other datasets with shorter reads.

Following the STARsolo documentation to match Cell Ranger results, for the pancreas and retina datasets, we used the following parameters for single-end alignment of read 2 to the mouse reference indices and ensuing quantification: --readFilesIn path/to/read2.fastq.gz path/to/read1.fastq.gz --soloType CB_UMI_Simple --clipAdapterType CellRanger4 --outFilterScoreMin 30 --soloCBmatchWLtype 1MM_multi_Nbase_pseudocounts --soloUMIfiltering MultiGeneUMI_CR --soloUMIdedup 1MM_CR --soloCellFilter EmptyDrops_CR --soloStrand Forward --soloBarcodeReadLength 0 --soloCBstart 1 --soloCBlen 16 --soloUMIstart 17 --soloUMIlen 10 --soloFeatures Gene Velocyto --readFilesCommand zcat.

In contrast, we performed paired-end alignment to the human reference index for the B cell dataset and for the samples from the T cell dataset with shorter read length (refer to Methods subsection Alevin-fry for details about read lengths). Based on the STARsolo documentation, this was achieved using the following parameters: --readFilesIn path/to/read1.fastq.gz path/to/read2.fastq.gz --soloType CB_UMI_Simple --outFilterScoreMin 30 --soloCBmatchWLtype 1MM_multi_Nbase_pseudocounts --soloUMIfiltering MultiGeneUMI_CR --soloUMIdedup 1MM_CR --soloCellFilter EmptyDrops_CR --soloBarcodeMate 1 --clip5pNbases 39 0 --soloCBstart 1 --soloCBlen 16 --soloUMIstart 17 --soloUMIlen 10 --soloFeatures Gene Velocyto --readFilesCommand zcat. Here, the read order was reversed compared to the input for the single-end alignment. When the same approach was used for the T cell samples with slightly longer reads, STARsolo crashed with a segmentation fault error. Therefore, we used single-end alignment for those samples with the following arguments: --readFilesIn path/to/read2.fastq.gz path/to/read1.fastq.gz --soloType CB_UMI_Simple --outFilterScoreMin 30 --soloCBmatchWLtype 1MM_multi_Nbase_pseudocounts --soloUMIfiltering MultiGeneUMI_CR--soloUMIdedup 1MM_CR --soloCellFilter EmptyDrops_CR --soloStrand Reverse --soloBarcodeReadLength 0 --soloCBstart 1 --soloCBlen 16 --soloUMIstart 17 --soloUMIlen 10 --soloFeatures Gene Velocyto --readFilesCommand zcat.

Two main differences between the paired- and single-end alignment of the 5’ data are the order of the read files and the parameter soloStrand. Because of the reversed order when performing paired-end alignment, soloStrand needs to be set to “Forward” here to ensure correct read orientation: While read 2 has an antisense orientation, the aligned portion of read 1 is on the sense strand relative to the reference transcriptome.

For all datasets, we used the Cell Ranger 737K barcodes as the whitelist, matching the 10x Genomics assay chemistry. An overview of the parameter settings is shown in Table 6.

**Table 6:** Parameter settings for STARsolo. For the pancreas, retina, and B cell datasets, the same parameters were used for all samples. For the T cell dataset, different settings were used for samples where Cell Ranger performed single-end (SE) and paired-end (PE) alignment. For the parameter readFilesIn, the path to the corresponding FASTQ file for read 1 and read 2 is indicated by R1 or R2, respectively. An asterisk (*) marks settings that correspond to the default value and were not explicitly set.

Run Mode	Parameter	Pancreas	Retina	B cells	T cells	
		all	all	all	SE	PE
genomeGenerate	sjdbOverhang	149	100	100	100	100
alignReads	readFilesIn	〈R2〉 〈R1〉	〈R2〉 〈R1〉	〈R1〉 〈R2〉	〈R1) 〈R2〉	〈R2〉 〈R1〉
	soloType	CB_UMI_Simple				
	clipAdapterType	CellRanger4				
	outFilterScoreMin	30				
	soloCBmatchWLtype	1MM_multi_Nbase_pseudocounts				
	soloUMIfiltering	MultiGeneUMI_CR				
	soloUMIdedup	1MM_CR				
	soloCellFilter	EmptyDrops_CR				
	soloBarcodeMate	0*	0*	1	1	0*
	clip5pNbases	0*	0*	39 0	39 0	0*
	soloStrand	Forward	Forward	Forward	Forward	Reverse
	soloBarcodeReadLength	0	0	1*	1*	0
	soloCBstart	1				
	soloCBlen	16				
	soloUMIstart	17				
	soloUMIlen	10				
	soloFeatures	Gene Velocyto				
	readFilesCommand	zcat				

### Tidesurf

For quantification of spliced and unspliced transcripts with tidesurf (v0.2.0), the Cell Ranger output directories were processed using default parameters and the respective gene annotation GTF files from the Cell Ranger references. For 3’ data, the orientation was specified as sense, while it was set to antisense for 5’ datasets.

### Data processing and RNA velocity analysis

All datasets were processed using scanpy (v1.11.1) [30] and scVelo (v0.3.3) [10]. For comparisons of counts per cell (total or splice state-specific), the processed reference datasets and the count matrices obtained from Cell Ranger, velocyto, alevin-fry, and tidesurf were filtered to contain only common cells (Table 7).

**Table 7:** Cell numbers in the analyzed datasets.

		Number of cells	
Dataset	Publication	Reference	Common
Pancreas	Bastidas-Ponce *et al.* [19]	3,696	3,696
Retina	Lo Giudice *et al.* [20]	2,726	2,635
B cells	Stewart *et al.* [11]	8,771	7,310
T cells	Fu *et al.* [13]	51,504	50,314

For downstream RNA velocity analysis, the datasets were additionally subsetted to common genes. Genes with fewer than 20 counts (spliced and unspliced) were filtered out. The gene expression matrix, as well as the spliced and unspliced counts, were normalized so that the total counts per cell were equal to the median total counts before normalization. Highly variable genes were determined with scvelo.pp.filter_genes_dispersion, using the seurat flavor and the top 2,000 genes. The expression matrix was log(x+1)-transformed. The first 30 principal components from the respective reference dataset were used to compute a neighborhood graph with 30 neighbors. Based on this neighborhood graph, moments of spliced and unspliced counts were calculated, and velocities were computed using scVelo’s stochastic model. Finally, velocities were projected onto the 2-dimensional UMAP embedding of the respective reference dataset for visualization.

### PAGA

Partition-based graph abstraction (PAGA) [24] with velocity-directed edges was performed with the function scvelo.tl.paga on the processed data containing RNA velocity information using default parameters, except for disabling the minimum spanning tree pruning step. As described above (see Methods subsection Data processing and RNA velocity analysis), the same neighborhood graph was used for all quantification methods. The cell type annotations of the respective dataset were used as groups for PAGA.

In contrast to the other three datasets, the T cell dataset was subsetted to five clusters (c01, c02, c03, c04, and c07) used for PAGA trajectory inference by the original authors [13]. The neighborhood graph with 30 neighbors was recomputed from the reference dataset. Based on this neighborhood graph, moments of spliced and unspliced counts and velocities were computed as described before (see Methods subsection Data processing and RNA velocity analysis). Then, PAGA was run as described above.

## Supporting information

S1 FigCorrelation of Cell Ranger and velocyto counts of overlapping genes.(A) Relation between Cell Ranger counts and velocyto unspliced counts of overlapping genes on opposite strands for the T cell dataset [13]. Color indicates the Cell Ranger alignment mode for the corresponding sample (single-end (SE) or paired-end (PE)). $\rho_{SE}/\rho_{PE}$: Pearson correlation for SE and PE aligned samples, respectively. Genes on the x-axis are sorted in descending order of the proportion of their nucleotides overlapping with a gene located on the antiparallel strand (displayed on the y-axis). (B) Illustration of the overlap of the gene *MIF* on the forward strand with an exon of the long non-coding RNA *MIF-AS1* on the reverse strand. Grey arrows represent introns and indicate the direction of the respective transcripts. Colored boxes represent exons, split into coding DNA sequence (CDS, darker shades) and untranslated regions (UTR, lighter shades). (C-D) Relation between Cell Ranger counts for *MIF* and velocyto spliced counts for the overlapping transcript *MIF-AS1* for the B cell (C) and T cell (D) datasets. Color in subpanel D represents the Cell Ranger alignment mode for the corresponding sample (single-end (SE) or paired-end (PE)).(TIF)

S2 FigNo co-expression of overlapping genes.Relation between Cell Ranger counts of overlapping genes on opposite strands for the B cell dataset [11] (ρ: Pearson correlation). Genes on the x-axis are sorted in descending order of the proportion of their nucleotides overlapping with a gene located on the antiparallel strand (displayed on the y-axis). The same gene pairs as in Fig 1G are shown.(TIF)

S3 Fig Comparison of spliced, unspliced, and ambiguous counts across methods. (A-C) Boxplots of the difference from tidesurf for total spliced (A), unspliced (B), and ambiguous (C) counts per cell. (D-F) Boxplots of Pearson correlation of spliced (D), unspliced (E), and ambiguous (F) counts per gene between methods.
(TIF)

S4 Fig Comparison of RNA velocities between methods. (A-B) Visualization of velocities for the developing retina (A) [20] and T cell (B) [13] datasets, computed from spliced and unspliced counts obtained with velocyto, alevin-fry, STARsolo, or tidesurf (top to bottom) on a two-dimensional UMAP embedding. In the top three subpanels, color represents the cosine similarity between two-dimensional UMAP velocity projections between the respective method and tidesurf. In the bottom panel, color represents cell type. (C) Boxplot of cosine similarity of velocity vectors per cell between methods for all genes. (D) Boxplot of Pearson correlation of velocities per gene between methods for all genes.
(TIF)

S5 Fig Comparison of inferred cluster transitions. (A-B) Partition-based graph abstraction (PAGA) [24] with velocity-directed edges for the developing retina [20] (A) and T cell [13] datasets (B). The underlying RNA velocity estimates were computed from the spliced and unspliced counts obtained with velocyto, alevin-fry, STARsolo, and tidesurf, respectively (top to bottom). Dashed lines show connectivities, and solid arrows indicate transitions.
(TIF)

S6 Fig Comparison of run time and memory requirements. (A-B) Mean run time (A) and utilized memory (B) for five selected samples, plotted against the number of reads in the sample. The lines represent the mean of five runs. Error bars show standard deviation.
(TIF)
