# Supplementary material for: Accurate quantification of spliced and unspliced transcripts for single-cell RNA sequencing with tidesurf
Source: PLoS One. 2026 Aug 17;21(8):e0355867. doi: 10.1371/journal.pone.0355867 (PMC13480657; doi:10.1371/journal.pone.0355867)
Supplement: Supplementary file 1 [file pone.0355867.s009.pdf]

Please find below the response to the reviewer comments. Reviewer comments are copied in green font, author responses in black font, and text citations from the revised manuscript are displayed in black italic font. Reference numbers within the cited text refer to the manuscript references.

Reviewer #1: The manuscript “Accurate quantification of spliced and unspliced transcripts for single-cell RNA sequencing with tidesurf” by Schleicher et al. describes the implementation of a new tool, tidesurf, for RNA velocity inference. The key advance of this tool is its ability to infer RNA velocity measures from both 3′- and 5′-based scRNA-seq protocols. The authors demonstrate clear advantages of tidesurf over velocyto, which was not designed to handle 5′-based scRNA-seq protocols but has nevertheless been used for this purpose, often leading to misleading results. They present a strong and defensible case for the superiority of tidesurf in analyzing 5′-based scRNA-seq data by identifying the underlying issues that affect velocyto’s performance.
The authors also compare tidesurf with alevin-fry and STARsolo, showing high concordance between these tools for 5′-based scRNA-seq protocols and demonstrating that all tools perform similarly on 3′-based scRNA-seq. The performance benchmarks provided in the supplementary materials are additionally helpful for understanding how these tools compare depending on what resources an investigator may have at their disposal.
One area where differences remain between tidesurf and alevin-fry is in datasets derived from human donors. The authors address this, noting: “The human genome contains more repetitive sequences than the murine genome [21], which could lead to differences in the (pseudo-) alignments of Cell Ranger and alevin-fry, especially for intronic sequences. Furthermore, the total unspliced counts were considerably lower on the two 5′ datasets, rendering small absolute differences relatively more important. Finally, alevin-fry’s quantification of total counts differed slightly more from the Cell Ranger output than tidesurf’s (Fig. 3B–D).”
I wonder whether there is a dataset that could serve as a ground truth (even a simulated dataset) to more directly evaluate the performance of these tools and clarify the sources of similarity or divergence between tidesurf and alevin-fry. One possible candidate is the dataset described in PMID: 38782298, which includes both long-read and short-read data generated from the same 10x Chromium samples. Although this dataset is not human, and the authors suggest the discrepancies may be human-specific, it could still provide informative insights for readers seeking to understand how these tools behave under conditions where transcript structures are more fully resolved.

We thank the reviewer for the suggestion. Unfortunately, to the best of our knowledge, the currently available scRNA-seq read simulators such as scReadSim (Yan et al. 2023, https://doi.org/10.1038/s41467-023-43162-w) do not generate separate ground-truth count matrices for spliced and unspliced molecules, and are thus not suited for our case. We looked into the dataset mentioned by the reviewer as an alternative. However, the authors of the study “Systematic evaluation of single-cell RNA-seq analyses performance based on long-read sequencing platforms” (Deng et al. 2025, https://doi.org/10.1016/j.jare.2024.05.020) did not make their data publicly available. We found another, similar dataset with paired long- and short-read sequencing data for human samples (Zajac et al. 2025, https://doi.org/10.1093/nargab/lqaf089) and obtained all necessary data from the publicly available repository and by direct request to the authors.

In the following, we describe

1. Details of data quantification for both short-read and long-read data
2. Comparison of total/unspliced counts
3. Gene-wise count correlation
4. Comparison of isoform category assignments

1. **Details of data quantification for both short-read and long-read data**

For quantification of the short-read data with Cell Ranger (v7.1.0), we first generated a Cell Ranger-compatible reference from the human reference genome GRCh38.p13 reference genome with GENCODE v39 annotations, obtained from SMRT Link (v13.1). Then, we mapped the short reads to the reference with Cell Ranger count. We filtered the resulting BAM files and the mapped long-read BAM files that we obtained from the authors to reads corresponding to cell barcode/unique molecular identifier (UMI) combinations that were present in both. For the short reads, we generated FASTQ files from the filtered BAM files with bamtofastq, realigned and quantified them with Cell Ranger. In contrast to the original study, we included intronic reads in the quantification. We used velocyto (v0.17.17) and tidesurf (v0.3.0) with default parameters (except for cell filtering, which we deactivated) to obtain splice-state-specific quantifications. For tidesurf, we also saved intermediate results in the form of tables containing the assigned gene and splice state for each UMI (one table per cell barcode). Additionally, we created a spliced + intronic reference with pyroe (v0.9.3) make-splici from the reference genome mentioned above, with a read length of 91 (corresponding to the short reads) and a flank trim length of 5, and created an index with salmon (v1.10.2) index. The FASTQ files were quantified based on this reference and index with alevin-fry (v0.8.2) as described in our manuscript, using the parameter settings for 3' data.

For the long-read data, we quantified isoforms using the Iso-Seq pipeline within SMRT Link as described in the original study, starting from the mapped, filtered BAM files.

A bash script performing all steps described above for the quantification of short and long reads is available on the tidesurf projects GitHub repository (https://github.com/janschleicher/tidesurf_projects/blob/6ac2d00c3c06ccf9a2cb0731de1f4a9fab765924/scripts/run_zajac.sh).

We processed all quantification results with scanpy (v1.11.1). For Cell Ranger, we used the unfiltered count matrix. For the long-read data, we separately used both the cell-by-gene and the cell-by-isoform count matrices. Since the cell-by-gene count matrix contained duplicated genes, we aggregated the counts per gene ID. The cell-by-isoform count matrix was summarized into the categories "spliced" and "unspliced" according to the following criteria, based on the pigeon isoform categories (https://isoseq.how/classification/categories.html, accessed 15.06.2026), as follows: isoforms falling into the categories "antisense", "intergenic", "fusion", or "moreJunctions" were removed. Counts from the category "genic intron" as well as counts for the category "genic" corresponding to isoforms with more than 1 reference exon (or an unknown number) were mapped to unspliced. All other categories ("full splice match", "incomplete splice match", "genic" with a single exon, "novel in catalog", and "novel not in catalog") were considered spliced.

2. **Comparison total/unspliced counts**

All AnnData objects were filtered to contain only common cells, dropping 16 cells that were not contained in the velocyto quantification. First, we compared the total counts per cell, using the cell-by-gene count matrix for the long read data (Rebuttal Figure 1). While the distribution of total counts for all quantified genes was similar between the long-read data ("pacbio" in all figure legends) and all short-read-based quantifications, the total counts for the long reads were considerably lower when only common genes were considered (removing, e.g., any novel or fusion genes identified from the long reads). This was also visible in the considerably higher difference per cell between the long-read data and Cell Ranger compared to any other quantification method for common genes.

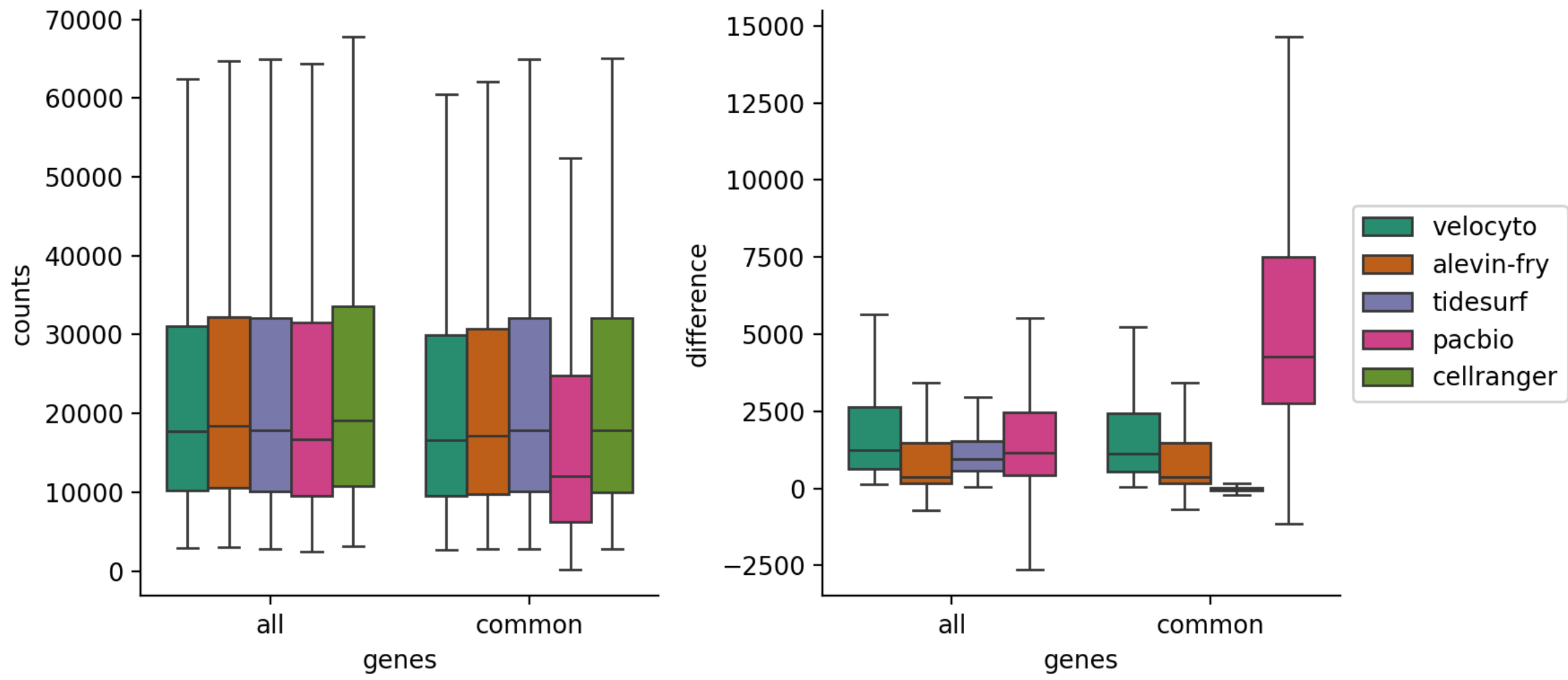

Rebuttal Figure 1: Left: boxplot of total counts per cell (sum of spliced, unspliced, and ambiguous for velocyto, alevin-fry, and tidesurf). Right: boxplot of differences of total counts per cell from Cell Ranger counts.

This difference in total counts between the long-read and short-read quantifications is mainly due to a difference in the unspliced counts (Rebuttal Figure 2). In this analysis, we added the ambiguous counts to the spliced counts since there is no ambiguity for the long reads, and this is the most likely origin of the ambiguous counts. On average, for the long read data, unspliced molecules account for around 6 % of all counts, while they represent around 20 % of the total counts for the other three methods (Rebuttal Figure 3).

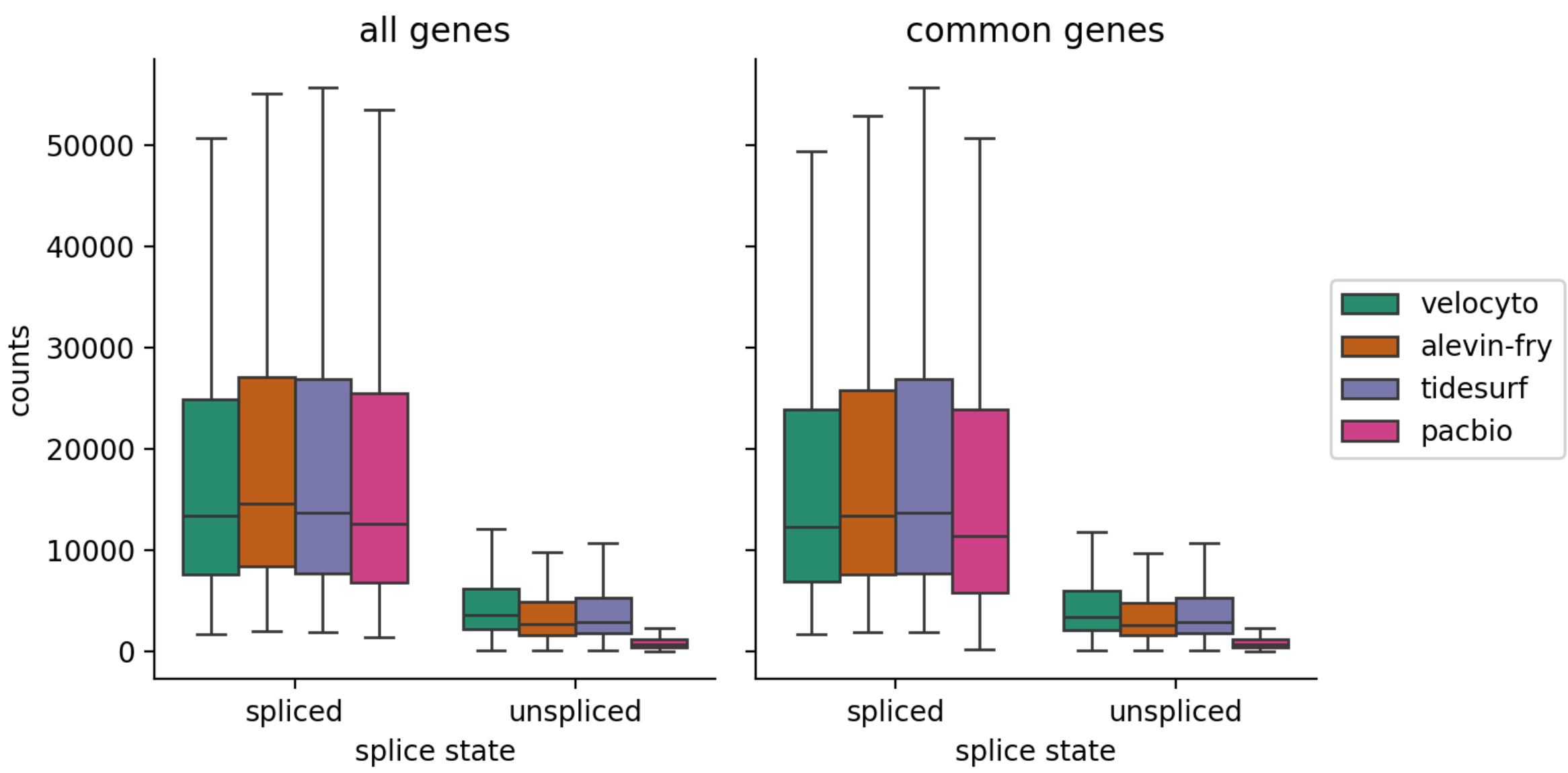

Rebuttal Figure 2: Boxplots of total spliced and unspliced counts per cell for all (left) and common (right) genes.

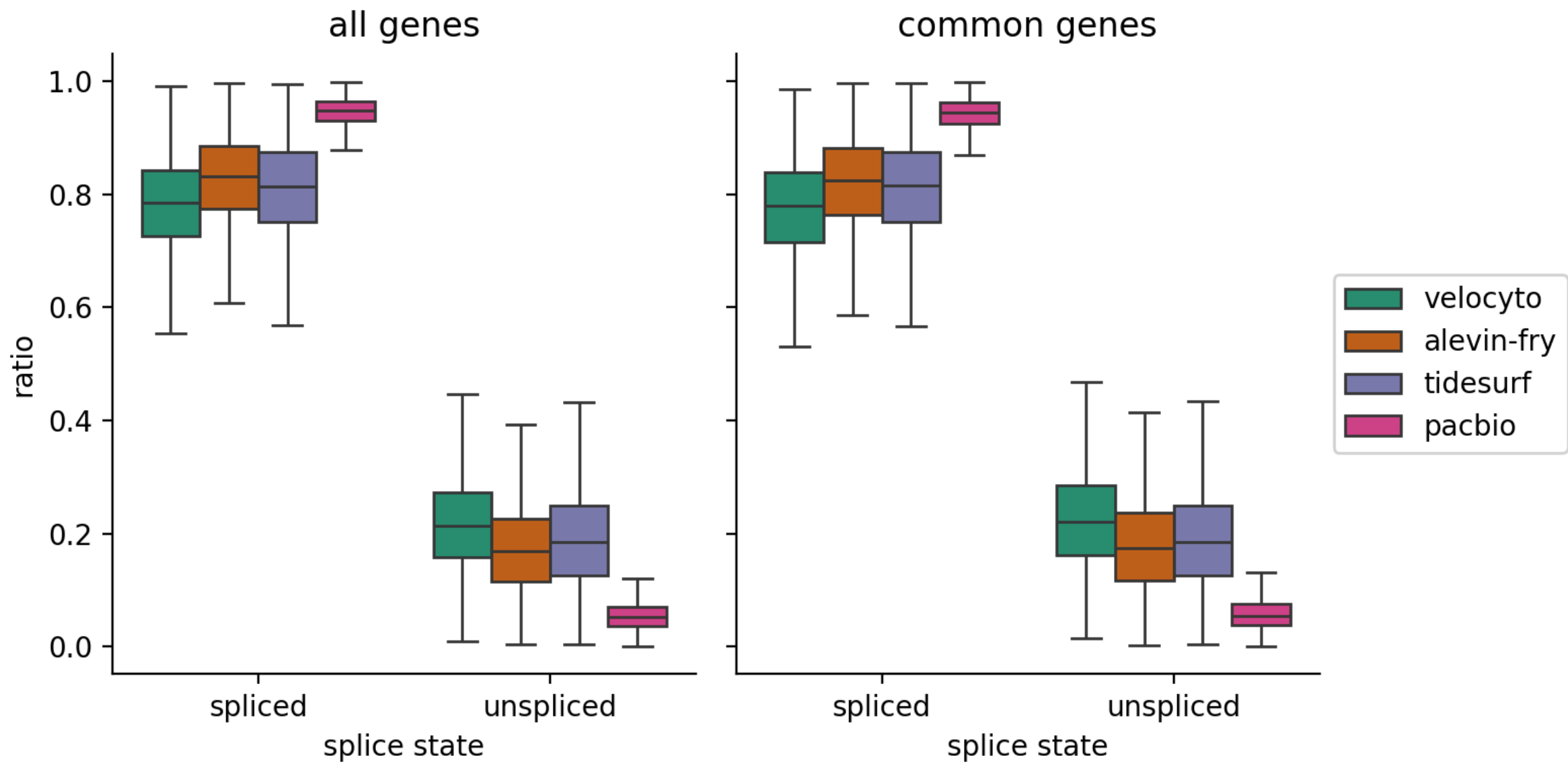

Rebuttal Figure 3: Boxplots of ratios of spliced and unspliced counts out of all counts per cell for all (left) and common (right) genes.

3. Gene-wise count correlation

Accordingly, it is not surprising that the gene-wise Pearson correlation (Rebuttal Figure 4) and cell-wise cosine similarity (Rebuttal Figure 5) between the long-read and short-read quantifications are lower for unspliced counts than for spliced counts. With respect to the Pearson correlation, there is no big difference between the three short-read quantification methods regarding their similarity to the long-read counts. For the cosine similarity, however, velocyto has a lower similarity to the long-read data for both spliced and unspliced counts than alevin-fry or tidesurf.

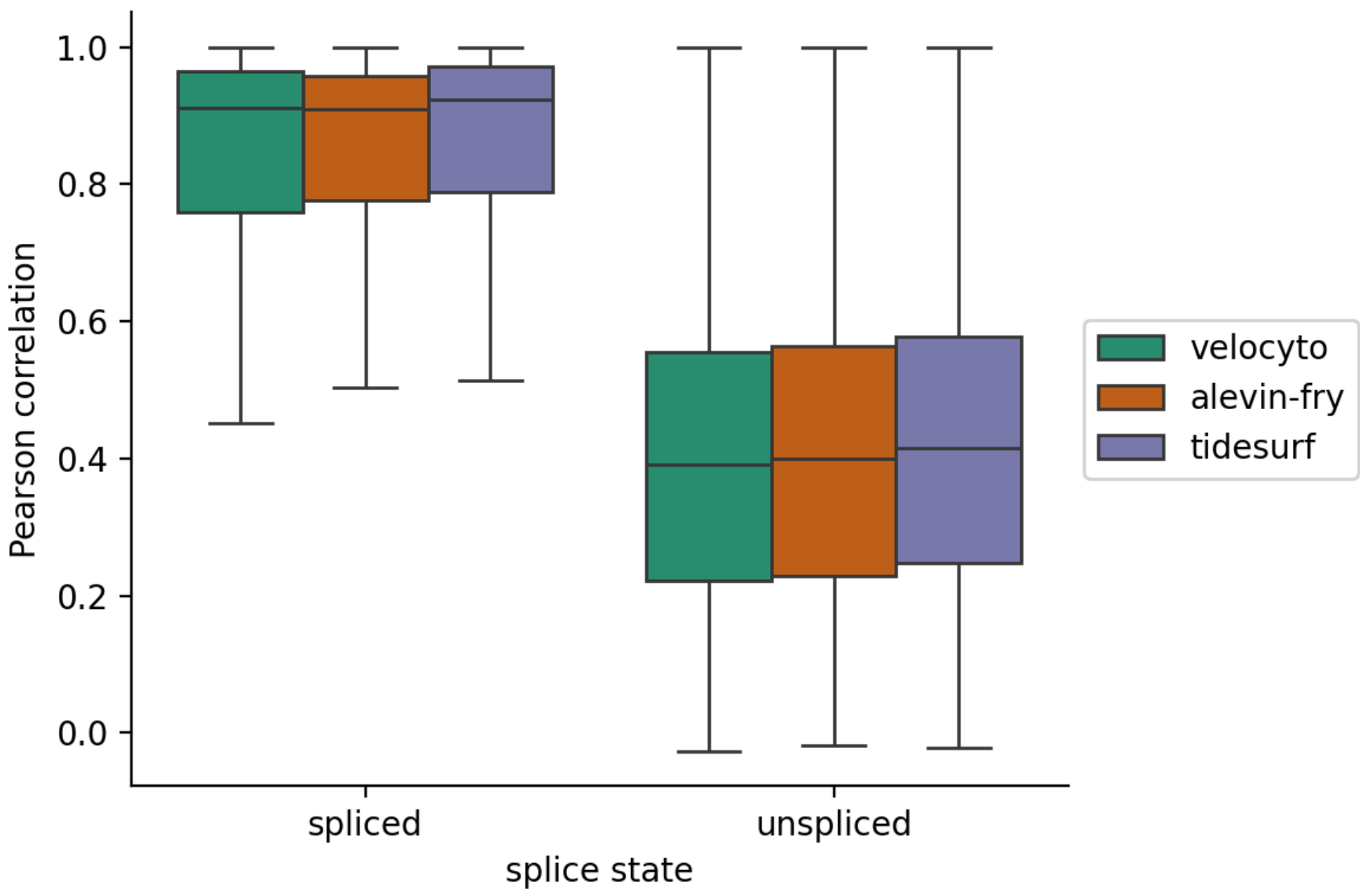

Rebuttal Figure 4: Boxplots of per-gene Pearson correlation of spliced and unspliced counts with the long-read data.

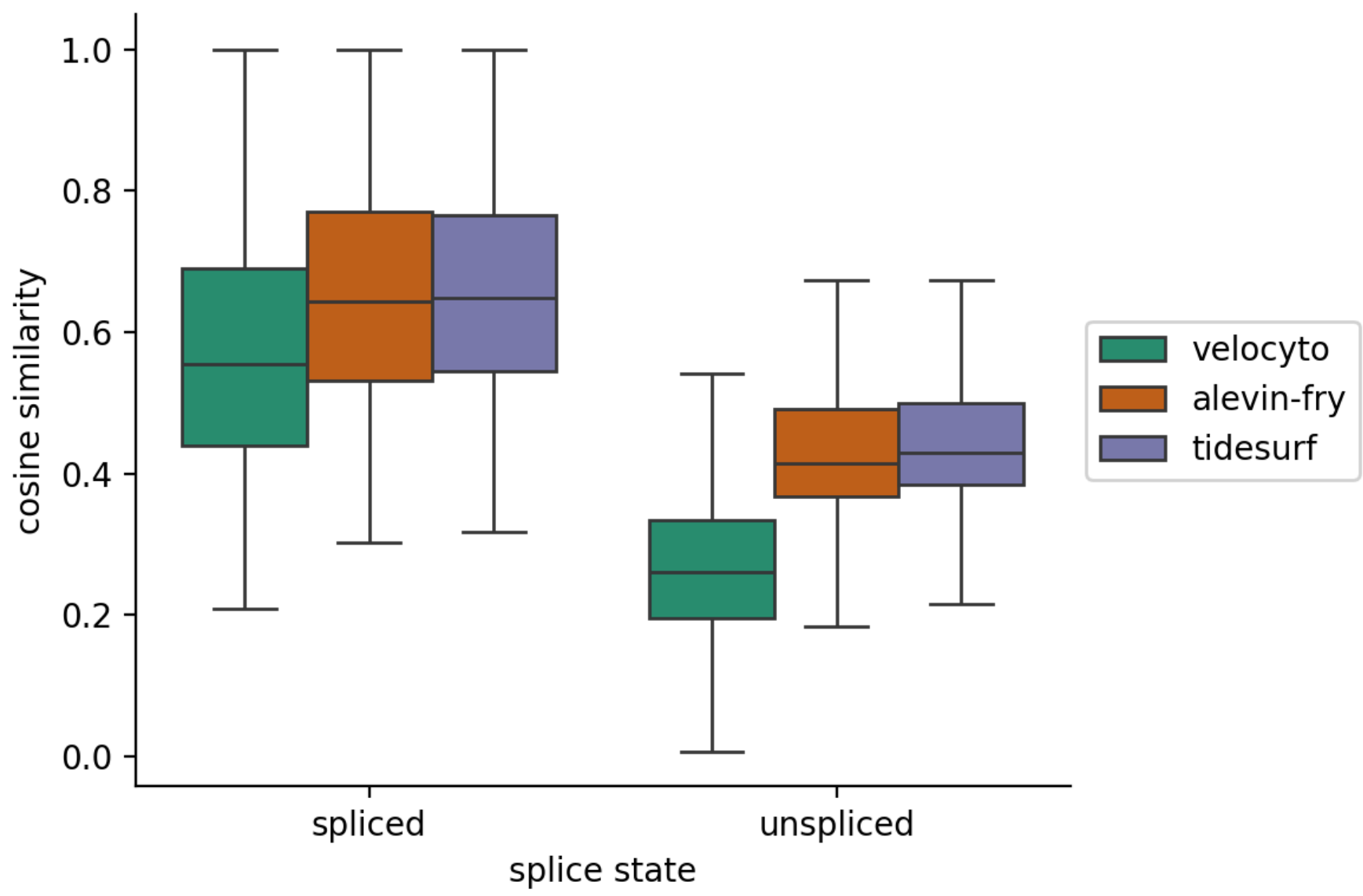

Rebuttal Figure 5: Boxplots of per-cell cosine similarity of spliced and unspliced counts with the long-read data.

4. Comparison of isoform category assignments

For tidesurf, we further investigated possible reasons for the observed discrepancies, in particular regarding the total unspliced counts. To this end, we exported intermediate results of the quantification in the form of tables with the assigned gene and splice state for each cell barcode and UMI, as described above. This allowed a direct comparison with the categories classified by the Iso-Seq pipeline for the same molecules by calculating a contingency matrix. The vast majority of all molecules were considered a full splice match by Iso-Seq and spliced by tidesurf (Rebuttal Figure 6).

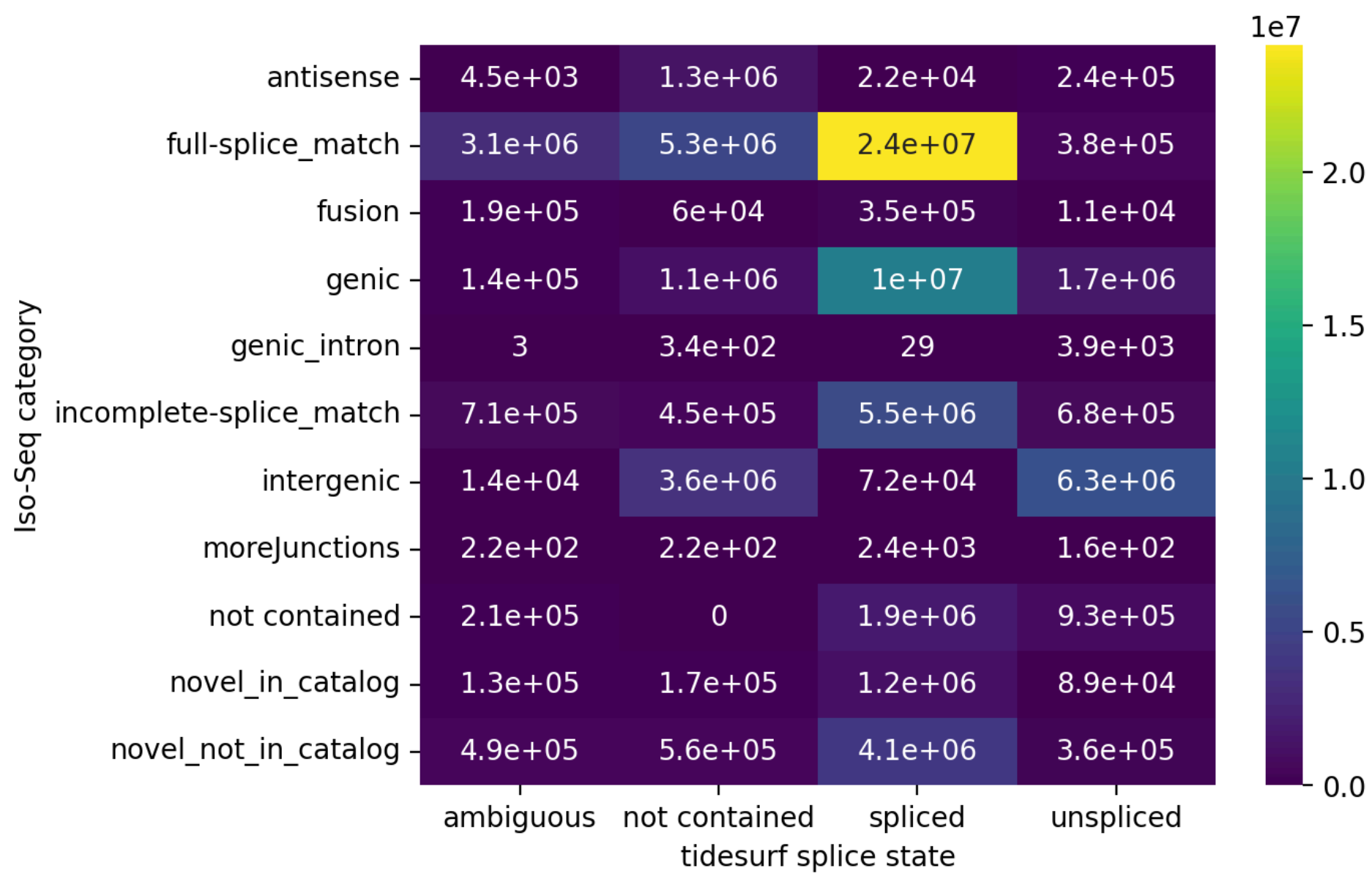

Rebuttal Figure 6: Contingency matrix of Iso-Seq isoform categories (y-axis) and tidesurf splice states (x-axis) for each molecule.

Relative to the number of molecules per Iso-Seq category (Rebuttal Figure 7, left), the majority of the molecules identified as full splice match, incomplete splice match, novel in

catalog, or novel not in catalog were classified as spliced by tidesurf, matching the expectation for this category. Similarly, the vast majority of molecules in the category "genic intron" were classified as unspliced. However, most of the molecules that tidesurf categorized as unspliced fell into the Iso-Seq class "intergenic" (Rebuttal Figure 7, right), and most of the "genic" molecules from Iso-Seq were classified as spliced.

The latter discrepancy is associated with single-exon genes, where Iso-Seq does not find splice junctions and therefore classifies the corresponding reads as genic. Rebuttal Figures 8 and 9 show long (genic) and short (spliced) reads aligned with the Iso-Seq pipeline and Cell Ranger, respectively, for the genes *IER5* and *HEXIM1* as representative examples, visualized with the integrative genome viewer (IGV) v2.19.7 on the GRCh38.p13 reference with GENCODE v39 annotations.

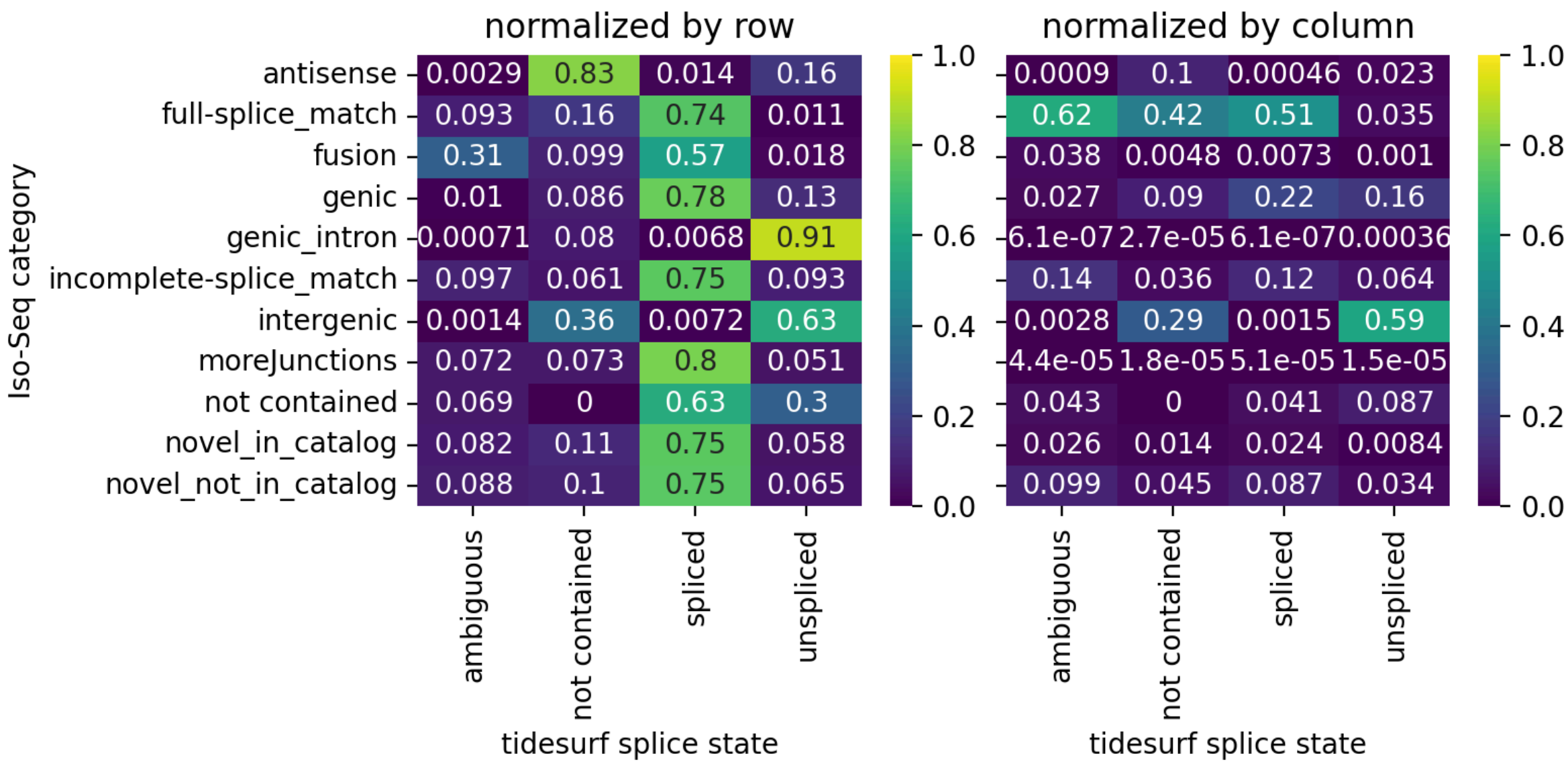

Rebuttal Figure 7: Contingency matrix of Iso-Seq isoform categories (y-axis) and tidesurf splice states (x-axis) for each molecule, normalized per Iso-Seq category (left) and tidesurf splice state (right).

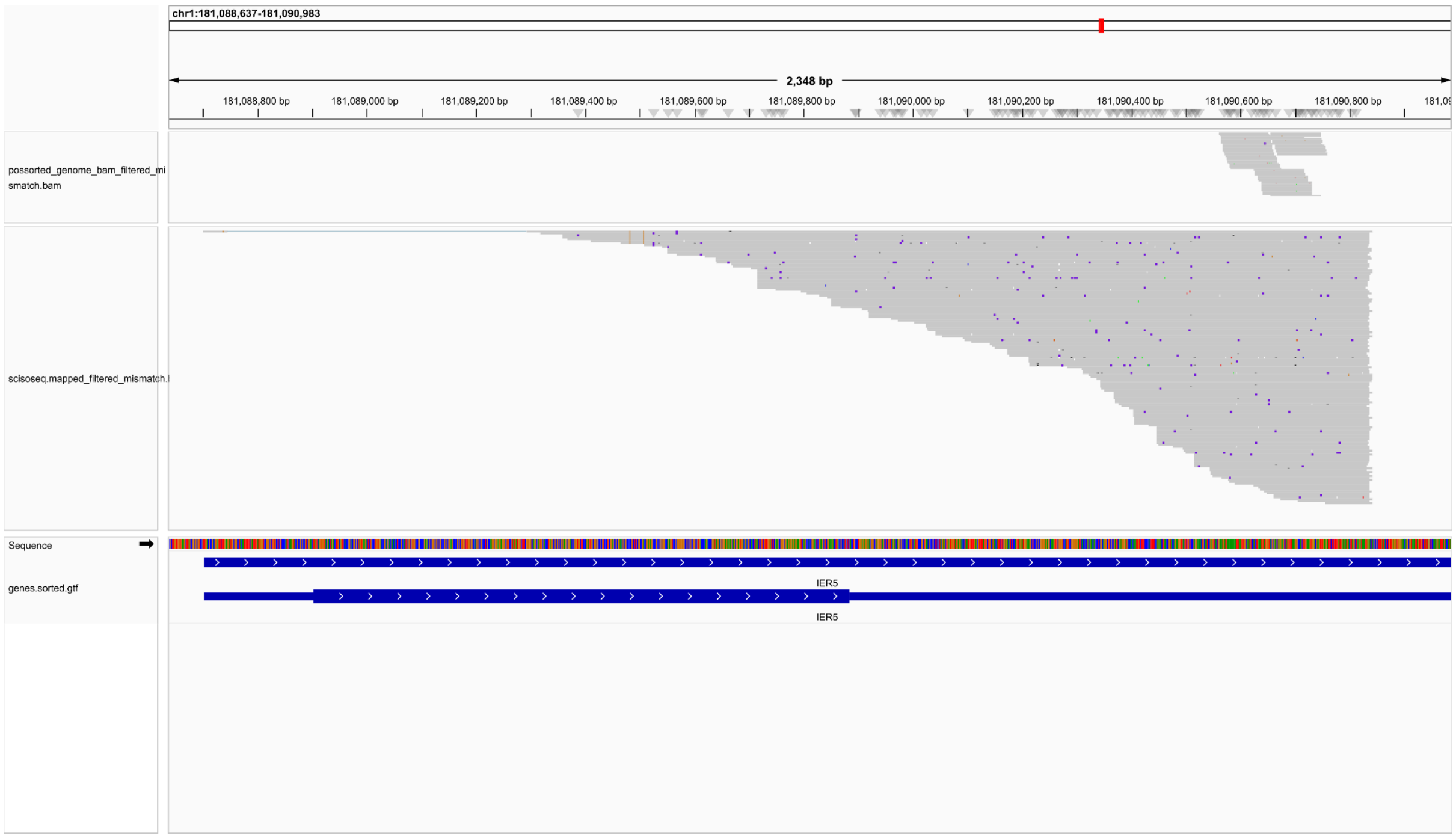

Rebuttal Figure 8: Read alignment for short (top) and long reads (bottom) for the *IER5* gene locus.

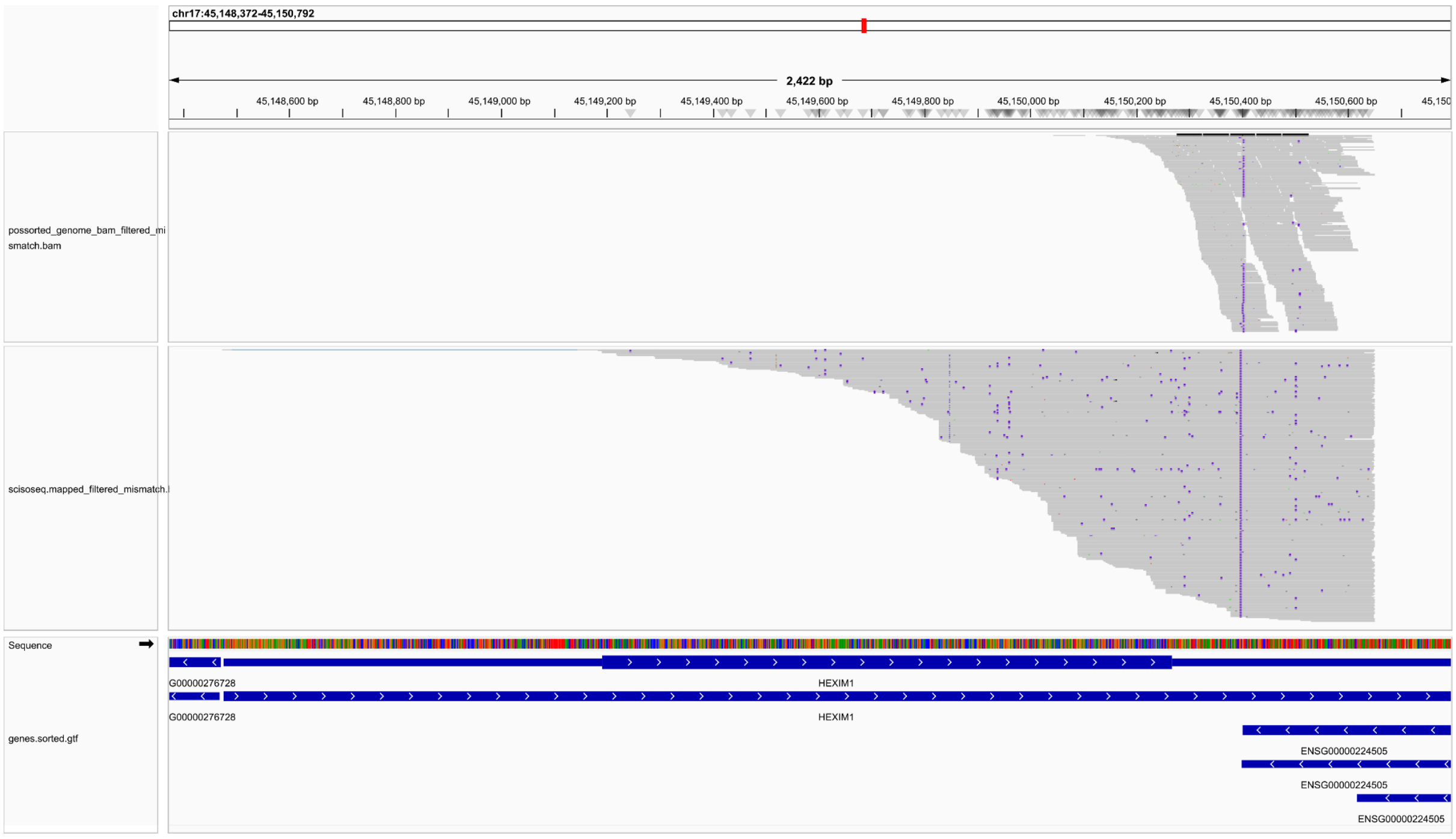

Rebuttal Figure 9: Read alignment for short (top) and long reads (bottom) for the *HEXIM1* gene locus.

For molecules that were identified as intergenic by Iso-Seq and as unspliced by tidesurf, we frequently observed that the corresponding short and long reads mapped exclusively to an intronic region according to the GENCODE v39 annotations, as exemplified by the genes *LRBA* (Rebuttal Figure 10) and *ELF2* (Rebuttal Figure 11). In this case, we do not know why they were classified as intergenic since, in our opinion, they represent unspliced molecules.

Finally, long reads classified as antisense, with corresponding unspliced short reads, frequently mapped to gene loci with overlapping genes. Here, Iso-Seq apparently chose the opposite-strand gene compared to Cell Ranger and tidesurf, and declared the molecule antisense to that gene, based on read orientation. For example, Cell Ranger and tidesurf assigned a molecule to the KDHC1 gene, which Iso-Seq categorized as antisense to KDHC1-AS1 (Rebuttal Figure 12). Similarly, molecules that Cell Ranger and tidesurf assigned to ENSG00000262198 were considered antisense to *LINC00621* by Iso-Seq (Rebuttal Figure 13). In these situations, we cannot know which of the two assignments is correct.

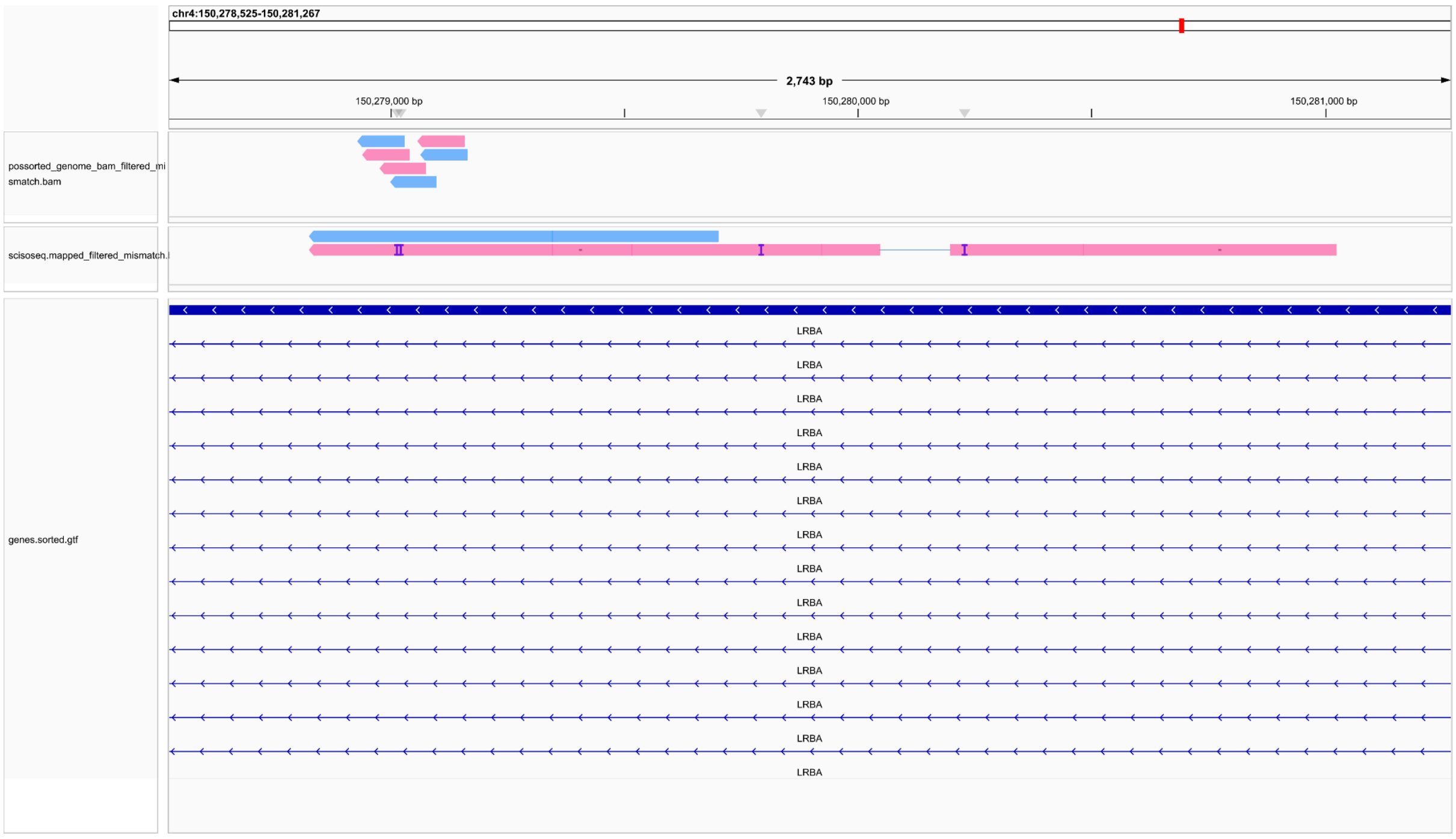

Rebuttal Figure 10: Read alignment for short (top) and long reads (bottom) for the *LRBA* gene locus. Reads stemming from the same molecule share the same color.

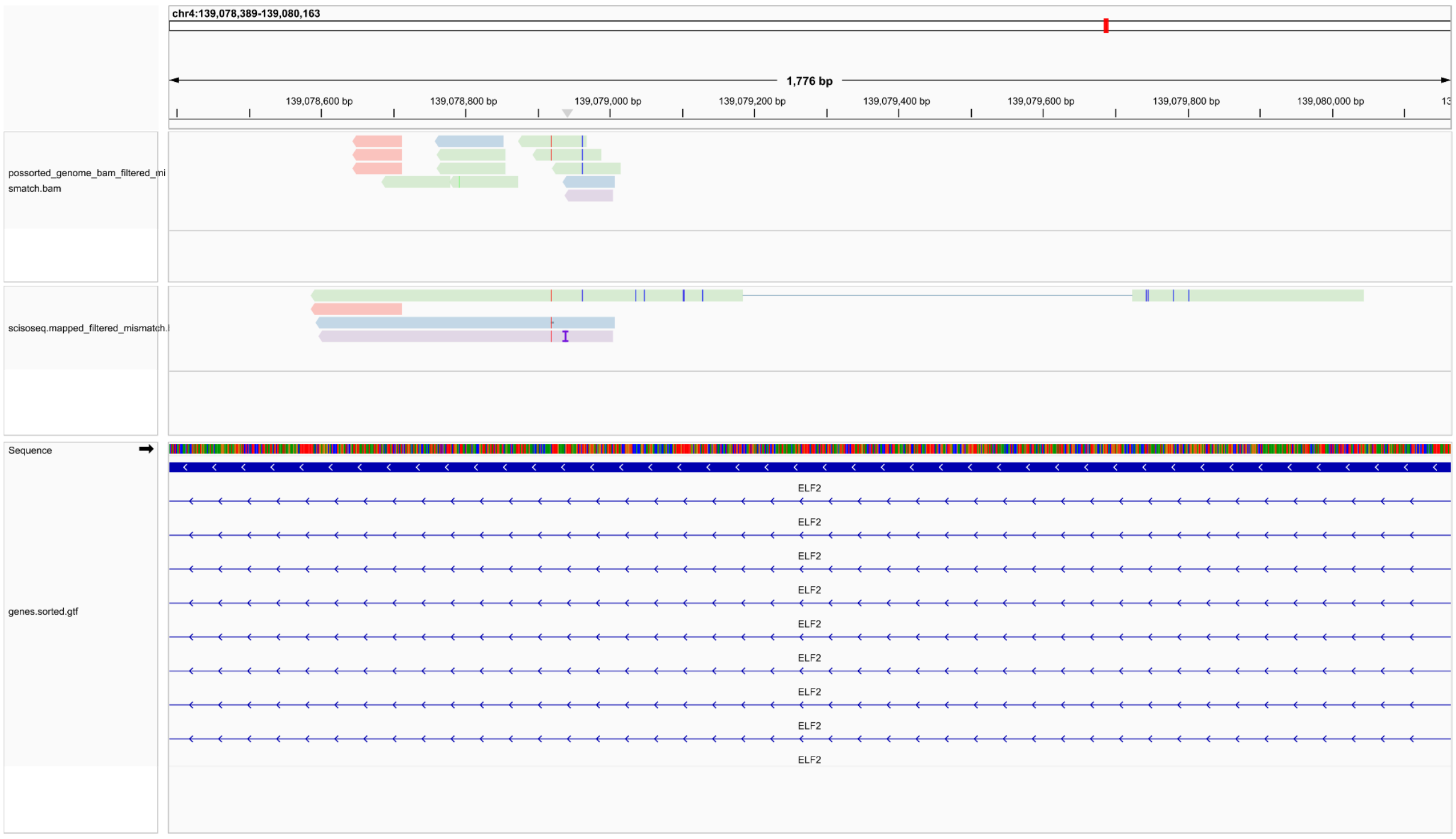

Rebuttal Figure 11: Read alignment for short (top) and long reads (bottom) for the *ELF2* gene locus. Reads stemming from the same molecule share the same color.

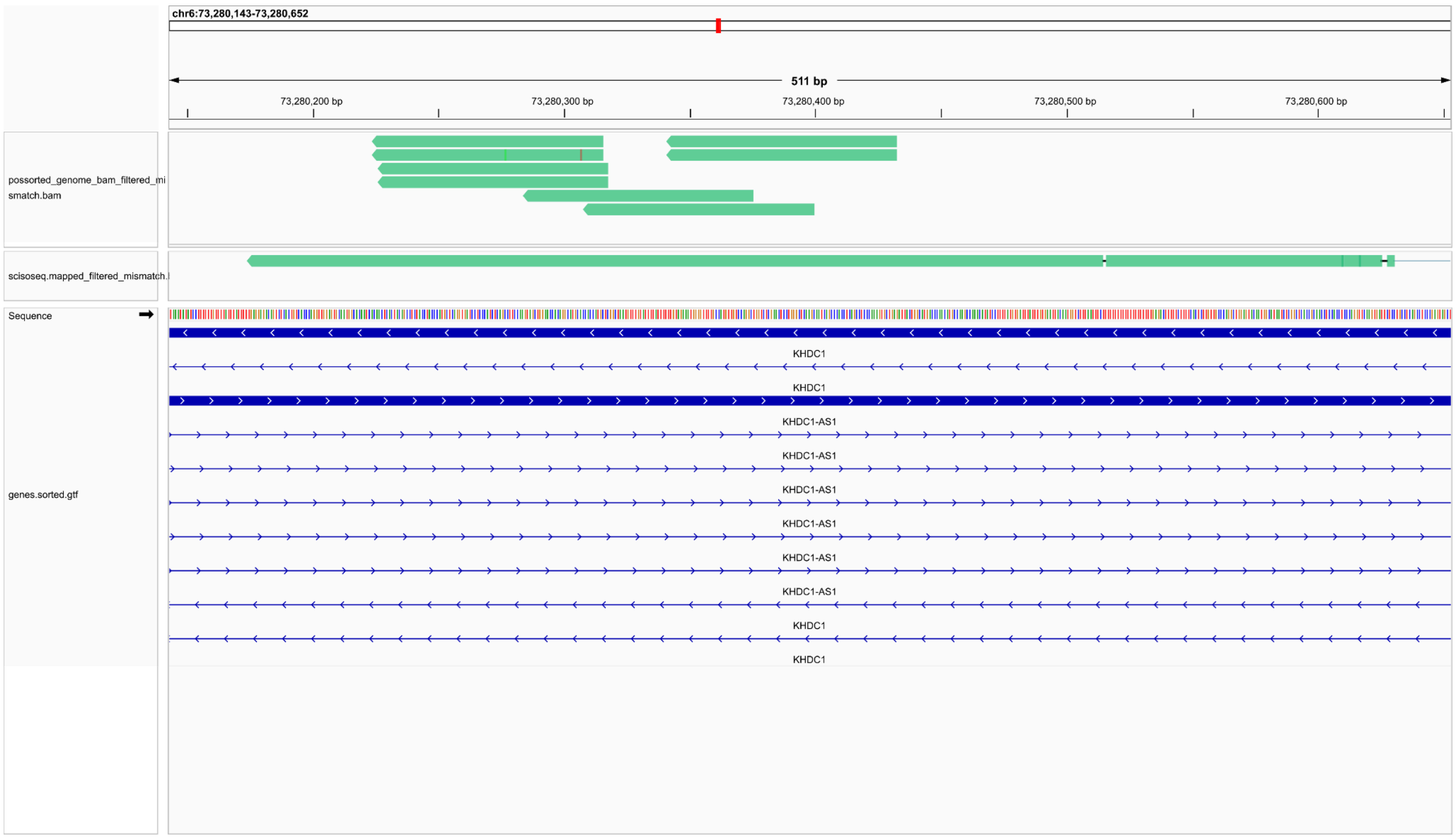

Rebuttal Figure 12: Read alignment for short (top) and long reads (bottom) for the *KDHC1/KDHC1-AS1* gene locus. Reads stemming from the same molecule share the same color.

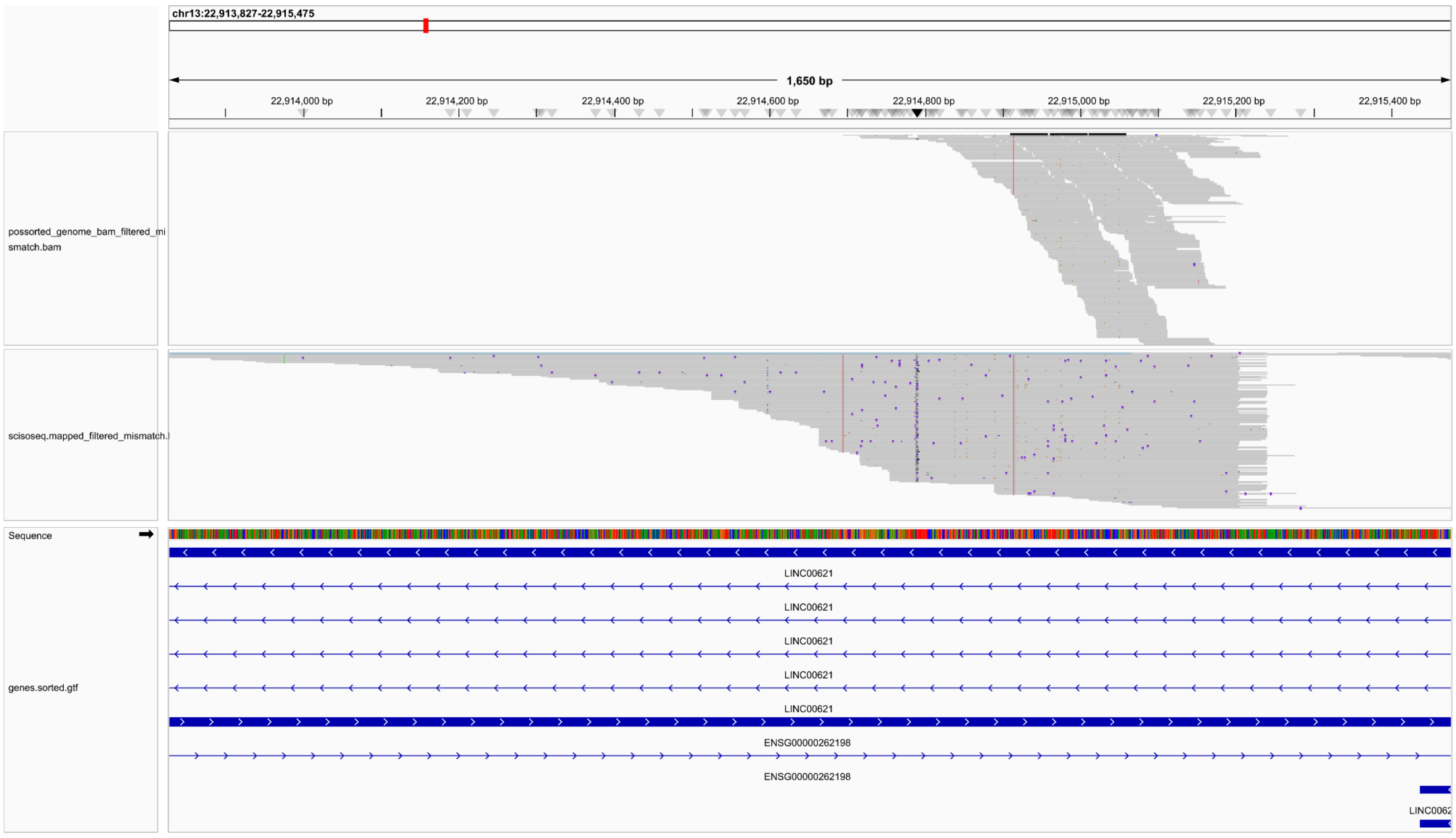

Rebuttal Figure 13: Read alignment for short (top) and long reads (bottom) for the *LINC00621/ENSG00000262198* gene locus.

In conclusion, with the current state of long-read single-cell RNA sequencing and the corresponding analysis workflow, we do not think that long-read data is a good proxy for ground truth splice state quantifications. Our analyses did not reveal major differences between alevin-fry, STARsolo, and tidesurf for this dataset, but highlighted that long- and short-read data cannot be properly compared, even when they sequence the same molecules. In particular, this is the case when introns are included in the Cell Ranger quantification: Zajac et al. (2025) showed higher concordance between short- and long-read-based counts, but did not include introns in their Cell Ranger quantification.

Reviewer #2: 1. Is the manuscript technically sound, and do the data support the conclusions?
Yes. The study is technically solid and the comparisons across datasets and tools are well done. The data generally support the main conclusion that velocyto performs poorly on 5′ scRNA-seq data, while tidesurf and other tools give more consistent results. The conclusions are reasonable based on the presented evidence.
2. Has the statistical analysis been performed appropriately and rigorously?
The analyses used (correlations, distribution comparisons, and velocity/PAGA comparisons) are appropriate for this type of benchmarking study. However, the statistical evaluation is mostly descriptive, and a bit more formal testing would improve clarity, although it is not critical.

We have implemented this suggestion by formally testing for differences between count distributions (total and per splice state) and ratio distributions per splice state with pairwise two-sided Mann-Whitney *U* tests. Furthermore, we tested whether total count differences from Cell Ranger and splice-state-specific count differences from tidesurf were significantly different from 0 with two-sided one-sample Student's *t*-tests. For all tests, we performed multiple testing correction with the Bonferroni method. Most tests were highly significant ($p < 0.001$), probably due to the large sample sizes (i.e., cell numbers). Therefore, we annotated non-significant differences in Fig. 3 and Fig. 4 with "ns" and added tables for all Mann-Whitney *U* test results (Tables 1-3). We updated the figure captions accordingly and referenced the tables in the main text.

3. Have the authors made all data underlying the findings fully available?
Yes. The datasets are clearly referenced with GEO/ArrayExpress accessions, and processed data and tools are described well enough to allow reproduction. Data availability is adequate.
4. Is the manuscript presented in an intelligible fashion and written in standard English?
Yes. The manuscript is clearly written and easy to follow. Only minor language polishing is needed in a few dense methodological sections.
5. Review comments to the author
The manuscript is well written and addresses an important issue in scRNA-seq velocity analysis. The findings are convincing and supported by multiple datasets and methods. T
A few minor points to improve the manuscript:
•The conclusions about "incorrect" assignments in velocyto could be slightly softened, since there is no direct ground truth.

We have softened the conclusions about incorrect assignments in velocyto to make it clearer that the results are unexpected given the Cell Ranger quantifications. As pointed out by the reviewer, we cannot say with certainty that velocyto's assignments are incorrect. There are, however, strong indications in favor of that hypothesis since we assume that Cell Ranger, the proprietary software by 10x Genomics for the analysis of their Chromium data, correctly identifies the experiment chemistry and takes its peculiarities into account.

Below, we cite passages from the manuscript where we softened statements concerning velocyto.

Abstract:

> *We demonstrate that velocyto systematically assumes opposite transcript direction compared to 10x Genomics Cell Ranger in 5'-sequencing data, leading to different count assignments than for other tools, substantial deviations in inferred velocities, and ultimately divergent biological interpretations. To address these shortcomings, we present tidesurf, a command-line tool designed for accurate quantification of spliced and unspliced molecules across both 3'- and 5'-based scRNA-seq protocols.*

By evaluating tidesurf on four publicly available 10x Genomics Chromium datasets and comparing it to state-of-the-art quantification approaches, we show that it reliably recovers correct transcript counts in settings where velocyto performs well (3' chemistry) and where it produces unexpected results (5' chemistry).

Introduction:
Lines 30-33:

This is potentially problematic as velocyto has not been updated since the beginning of 2019, despite the significant progress in scRNA-seq protocols. For example, it does not offer a mode for the analysis of 10x Genomics 5' scRNA-seq data, resulting in unexpected quantification results when used on such datasets.

Lines 37-40:

We show that, compared to Cell Ranger, velocyto severely underestimates the total number of transcripts and assigns gene counts to overlapping genes on the opposite strand, assuming a different orientation of sequencing reads.

Results:
Lines 79-80:

Taken together, these results demonstrate that velocyto produces unexpected results on 10x Genomics 5' scRNA-seq data, particularly for single-end aligned samples.

Lines 82-86:

Next, we investigated the mechanistic causes of these discrepancies. While velocyto assumes strand-specific reads, it does not take into account that the orientation of reads differs between 5' and 3' protocols. We hypothesized that the observed unspliced counts do not arise from the genes to which Cell Ranger assigns the reads, but rather from transcripts on the opposing DNA strand at the same position.

Lines 102-104:

As a result, it frequently did not assign reads to the same gene as Cell Ranger, but to another, overlapping gene on the opposite strand.

Lines 220-222:

As described above, the considerable differences between velocyto and the other three tools on the 5’ datasets were expected since velocyto’s quantification also differed considerably from Cell Ranger for these protocols (Fig 1, S1 Fig).

Discussion:
Lines 364-368:

By reanalyzing the data from two such studies, we demonstrated that velocyto assigned reads to overlapping genes on the opposite strand compared to Cell Ranger, 10x Genomics’ own software, for 5’ scRNA-seq data, resulting in considerably underestimated total counts and highly inflated unspliced proportions.

Lines 376-379:

Consequently, tidesurf represents a replacement for velocyto on 5’ data, where the latter produces count matrices that differ considerably from those produced by any other tool tested here.

Lines 420-423:

> *Because of this and because of the aforementioned assignment to transcripts on the opposite strand compared to Cell Ranger, we assume that the results of velocyto on the 5' datasets were less reliable than the results based on the other quantification tools.*

Conclusion:
Lines 434-441:

> *We demonstrate that velocyto, the seminal tool for quantification of spliced and unspliced transcripts from aligned scRNA-seq reads, systematically assumes a different read direction in 10x Genomics 5' scRNA-seq data than Cell Ranger, leading to the assignment of counts to overlapping transcripts, affecting RNA velocity estimates, downstream analyses, and their biological interpretation, with results differing from those obtained with other tools. Therefore, we advise against using velocyto on scRNA-seq data generated with recent experimental protocols, such as 10x 5' scRNA-seq.*

•A small summary table comparing tool settings would improve readability.

Since the methods subsections describing the parameters used for quantification with alevin-fry and STARsolo, respectively, were indeed hard to read, we added tables as an overview of the tool settings used for each dataset (Tables 5 and 6, on pages 20 and 22). With this addition, the values for all non-default parameters can be seen at a glance. We also added a short paragraph to explain the parameters "readFilesIn" and "soloStrand" in more detail and show how they relate to the correct read orientation for 5' data (lines 589-593):

> *Two main differences between the paired- and single-end alignment of the 5' data are the order of the read files and the parameter soloStrand. Because of the reversed order when performing paired-end alignment, soloStrand needs to be set to "Forward" here to ensure correct read orientation: While read 2 has an antisense orientation, the aligned portion of read 1 is on the sense strand relative to the reference transcriptome.*

Generally, this is a solid and useful methodological paper with clear practical relevance.

Reviewer #3: The manuscript talks about an interesting aspect of identifying spliced and unspliced RNA transcripts but I am little confused as to why the authors have downplayed Velocyto which looks like the crux of the entire manuscript. I understand that Velocyto has not been upgraded to include the pitfalls of 5' chemistry but what if they do that upgrade soon and make Velocyto as powerful as 10X genomics softwares.
It would have been nice if the authors have introduced Tidesurf as the new command line software which addresses the pitfalls of existing softwares such as Velocyto in a sentence or two but not make it the crux of the entire manuscript.
Can the existing manuscript be rearranged and rewritten so as to put the focus completely on the USPs of Tidesurf highlighting the real time importance rather than downgrading an existing software which was introduced in 2018-2019. I would say few sentences about Velocyto are in harsh language which is not desired for the readers.
If that modification (major revision) can be done than the manuscript will be in a good shape and can be considered for publication.

We acknowledge that our manuscript included some harsh statements about velocyto, which we toned down in the revised manuscript. In particular, as remarked by reviewer 2, in the absence of a ground truth, it is not possible to claim without a doubt that velocyto produces incorrect quantifications. Please refer to our response to reviewer 2 above to see how and where we softened corresponding statements in the manuscript.

Based on the discrepancies between the velocyto and Cell Ranger quantifications on 5' data, with Cell Ranger being a software suite provided by 10x Genomics for the analysis of data generated with their kits, and therefore suitable for any chemistry they provide, we believe, however, that velocyto makes mistakes that the other tools do not make. In particular, the high correlation between velocyto unspliced counts and Cell Ranger counts for genes on the opposite strand clearly indicates that velocyto, unlike the other tested tools, assigns reads to the opposite strand relative to Cell Ranger. While the Cell Ranger quantifications do not represent a ground truth, they consider the differing read orientation between 5' and 3' protocols and, therefore, are more likely correct than velocyto's assignments.

Regarding possible upgrades to velocyto, we would like to mention that the README on the velocyto GitHub repository explicitly mentions that it is not maintained anymore and that users should consider using alternatives such as STARSolo instead. We added this information to the manuscript (LINES 360-363):

> *Despite this technological progress, velocyto has not been updated, and on the velocyto GitHub repository (https://github.com/velocyto-team/velocyto.py, accessed 16.06.2026), the authors state that it is no longer maintained and should not be used anymore.*

In our manuscript, we want to position our own tool, tidesurf, as another possible alternative among a number of different options. It is easy to use for 10x Genomics data since the strand specificity for the respective protocols can be chosen simply by switching between the "sense" and "antisense" orientation of reads in relation to transcript definitions. For typical scRNA-seq workflows, where quantifications are performed with Cell Ranger and spliced/unspliced counts are added to the data later, it also provides the highest concordance with Cell Ranger count matrices. We highlighted the advantages of tidesurf in the discussion section (lines 383-407):

> *In contrast to alevin-fry and STARsolo, tidesurf can be more easily incorporated into standard pipelines where 10x Genomics Cell Ranger is commonly used for gene expression quantification. Tidesurf directly uses Cell Ranger's output and produces spliced, unspliced, and ambiguous counts for the same cell barcodes (or a superset if*

filtering is turned off) in convenient AnnData format for easy integration with Cell Ranger's expression matrix. For alevin-fry, on the other hand, it is necessary to run a second analysis from scratch, which can lead to the calling of slightly different cell barcodes. Similarly, STARsolo performs its own cell filtering steps, which may result in different cells than the Cell Ranger pipeline. While the STARsolo documentation, a single markdown document that is several versions behind the most recent release, describes parameter settings for obtaining similar results as with Cell Ranger, we still observed greater differences from Cell Ranger than when using tidesurf. Furthermore, the use of alevin-fry on Chromium 5' data requires the adjustment of two parameters relating to read orientation with respect to each other and the reference genome in two different functions, which we only detected through a thorough search of multiple issues on the respective project repositories. In the same way, we had to make multiple adjustments for STARsolo on the 5' data. Even with all adjustments made according to the documentation, when processing some samples with longer reads in paired-end alignment mode, STARsolo crashed with a segmentation fault, necessitating falling back to single-end alignment mode. This issue has been reported by many users on the STAR GitHub repository, which has almost 1,000 open issues. For tidesurf, only a single command line argument has to be changed to switch from the default sense orientation of reads to reference annotations for 3' data to the antisense orientation for 5' data. Moreover, the tidesurf quantification was closest to Cell Ranger across all tested datasets in terms of absolute count differences and expression profile similarity.

Furthermore, we do not wish to downplay velocyto. The work by La Manno et al. (2018) was seminal, introducing the concept of RNA velocity and, at the same time, providing the velocyto software to perform the required splice-state-specific quantifications and downstream velocity estimation. Nevertheless, even the authors of velocyto acknowledge that their software is outdated and should not be used anymore. At the same time, the documentation of scVelo (https://scvelo.readthedocs.io/en/stable/getting_started.html, accessed 16.06.2026), the current standard for RNA velocity estimation, to this day suggests generating the separate count matrices with velocyto. In the documentation of the velocyto CLI (https://velocyto.org/velocyto.py/tutorial/cli.html, accessed 02.06.2026), linked from the scVelo documentation, there is no similar warning as on the GitHub repository. Here, users can find the "run10x" command, which looks like it should work for any 10x Genomics Chromium dataset. We hypothesize that this is why many studies (see citations in the introduction of the manuscript, lines 33-34) used velocyto on 10x 5' data, and we want to caution against this practice with our work, since it can produce unexpected quantification results that are not in line with Cell Ranger and may lead to misleading interpretations of the downstream RNA velocity analysis. We have adjusted the manuscript accordingly to make this clearer (lines 434-441):

We demonstrate that velocyto, the seminal tool for quantification of spliced and unspliced transcripts from aligned scRNA-seq reads, systematically assumes a different read direction in 10x Genomics 5' scRNA-seq data than Cell Ranger, leading to the assignment of counts to overlapping transcripts, affecting RNA velocity estimates, downstream analyses, and their biological interpretation, with results differing from those obtained with other tools. Therefore, we advise against using velocyto on scRNA-seq data generated with recent experimental protocols, such as 10x 5' scRNA-seq.
