# Supplementary material for: Accurate quantification of spliced and unspliced transcripts for single-cell RNA sequencing with tidesurf
Source: PLoS One. 2026 Aug 17;21(8):e0355867. doi: 10.1371/journal.pone.0355867 (PMC13480657; doi:10.1371/journal.pone.0355867)
Supplement: Supplementary file 2 [file pone.0355867.s007.docx]

The manuscript *“Accurate quantification of spliced and unspliced transcripts for single-cell RNA sequencing with tidesurf”* by Schleicher et al. describes the implementation of a new tool, **tidesurf**, for RNA velocity inference. The key advance of this tool is its ability to infer RNA velocity measures from both 3′- and 5′-based scRNA-seq protocols. The authors demonstrate clear advantages of tidesurf over **velocyto**, which was not designed to handle 5′-based scRNA-seq protocols but has nevertheless been used for this purpose, often leading to misleading results. They present a strong and defensible case for the superiority of tidesurf in analyzing 5′-based scRNA-seq data by identifying the underlying issues that affect velocyto’s performance.

The authors also compare tidesurf with **alevin-fry** and **STARsolo**, showing high concordance between these tools for 5′-based scRNA-seq protocols and demonstrating that all tools perform similarly on 3′-based scRNA-seq. The performance benchmarks provided in the supplementary materials are additionally helpful for understanding how these tools compare depending on what resources an investigator may have at their disposal.

One area where differences remain between tidesurf and alevin-fry is in datasets derived from human donors. The authors address this, noting: “The human genome contains more repetitive sequences than the murine genome [21], which could lead to differences in the (pseudo-) alignments of Cell Ranger and alevin-fry, especially for intronic sequences. Furthermore, the total unspliced counts were considerably lower on the two 5′ datasets, rendering small absolute differences relatively more important. Finally, alevin-fry’s quantification of total counts differed slightly more from the Cell Ranger output than tidesurf’s (Fig. 3B–D).”

I wonder whether there is a dataset that could serve as a *ground truth* (even a simulated dataset) to more directly evaluate the performance of these tools and clarify the sources of similarity or divergence between tidesurf and alevin-fry. One possible candidate is the dataset described in **PMID: 38782298**, which includes both long-read and short-read data generated from the same 10x Chromium samples. Although this dataset is not human, and the authors suggest the discrepancies may be human-specific, it could still provide informative insights for readers seeking to understand how these tools behave under conditions where transcript structures are more fully resolved.
