# Supplementary material for: Accurate quantification of spliced and unspliced transcripts for single-cell RNA sequencing with tidesurf
Source: PLoS One. 2026 Aug 17;21(8):e0355867. doi: 10.1371/journal.pone.0355867 (PMC13480657; doi:10.1371/journal.pone.0355867)
Supplement: Peer review history

# Peer review: Decision Letter 0

**DOI:** 10.1371/journal.pone.0355867.r001

**Authors:** Andreas Neueder

4 May 2026

PONE-D-26-15640

Accurate quantification of spliced and unspliced transcripts for single-cell RNA sequencing with tidesurf.

PLOS One

Dear Dr. Claassen,

Thank you for submitting your manuscript to PLOS ONE. After careful consideration, we feel that it has merit but does not fully meet PLOS ONE’s publication criteria as it currently stands. Therefore, we invite you to submit a revised version of the manuscript that addresses the points raised during the review process.

The reviewers have suggested a few experiments and re-writes, which will strengthen the manuscript.

Please let me know if you need more time to conduct the revision.

Please submit your revised manuscript by Jun 18 2026 11:59PM. If you will need more time than this to complete your revisions, please reply to this message or contact the journal office at plosone@plos.org. When you're ready to submit your revision, log on to https://www.editorialmanager.com/pone/ and select the 'Submissions Needing Revision' folder to locate your manuscript file.

Please include the following items when submitting your revised manuscript:

- A letter that responds to each point raised by the academic editor and reviewer(s). You should upload this letter as a separate file labeled 'Response to Reviewers'.
- A marked-up copy of your manuscript that highlights changes made to the original version. You should upload this as a separate file labeled 'Revised Manuscript with Track Changes'.
- An unmarked version of your revised paper without tracked changes. You should upload this as a separate file labeled 'Manuscript'.

If you would like to make changes to your financial disclosure, please include your updated statement in your cover letter. Guidelines for resubmitting your figure files are available below the reviewer comments at the end of this letter.

If applicable, we recommend that you deposit your laboratory protocols in protocols.io to enhance the reproducibility of your results. Protocols.io assigns your protocol its own identifier (DOI) so that it can be cited independently in the future. For instructions see: https://journals.plos.org/plosone/s/submission-guidelines#loc-laboratory-protocols. Additionally, PLOS ONE offers an option for publishing peer-reviewed Lab Protocol articles, which describe protocols hosted on protocols.io. Read more information on sharing protocols at https://plos.org/protocols?utm_medium=editorial-email&utm_source=authorletters&utm_campaign=protocols.

As the corresponding author, your ORCID iD is verified in the submission system and will appear in the published article. PLOS supports the use of ORCID, and we encourage all coauthors to register for an ORCID iD and use it as well. Please encourage your coauthors to verify their ORCID iD within the submission system before final acceptance, as unverified ORCID iDs will not appear in the published article. *Only*  the individual author can complete the verification step; PLOS staff *cannot*  verify ORCID iDs on behalf of authors.

We look forward to receiving your revised manuscript.

Kind regards,

Andreas Neueder

Academic Editor

PLOS One

Journal Requirements:

When submitting your revision, we need you to address these additional requirements.

1. Please ensure that your manuscript meets PLOS ONE's style requirements, including those for file naming. The PLOS ONE style templates can be found at

https://journals.plos.org/plosone/s/file?id=wjVg/PLOSOne_formatting_sample_main_body.pdf and

https://journals.plos.org/plosone/s/file?id=ba62/PLOSOne_formatting_sample_title_authors_affiliations.pdf

2. We note that the grant information you provided in the ‘Funding Information’ and ‘Financial Disclosure’ sections do not match.

When you resubmit, please ensure that you provide the correct grant numbers for the awards you received for your study in the ‘Funding Information’ section.

3. Thank you for stating the following financial disclosure: [DFG CL 792/1-1, DFG EXC 2180].

Please state what role the funders took in the study.  If the funders had no role, please state: ""The funders had no role in study design, data collection and analysis, decision to publish, or preparation of the manuscript.""

If this statement is not correct you must amend it as needed.

Please include this amended Role of Funder statement in your cover letter; we will change the online submission form on your behalf.

4. Thank you for uploading your study's underlying data set. Unfortunately, the repository you have noted in your Data Availability statement does not qualify as an acceptable data repository according to PLOS's standards.

At this time, please upload the minimal data set necessary to replicate your study's findings to a stable, public repository (such as figshare or Dryad) and provide us with the relevant URLs, DOIs, or accession numbers that may be used to access these data. For a list of recommended repositories and additional information on PLOS standards for data deposition, please see https://journals.plos.org/plosone/s/recommended-repositories.

5. If the reviewer comments include a recommendation to cite specific previously published works, please review and evaluate these publications to determine whether they are relevant and should be cited. There is no requirement to cite these works unless the editor has indicated otherwise.

6. Please review your reference list to ensure that it is complete and correct. If you have cited papers that have been retracted, please include the rationale for doing so in the manuscript text, or remove these references and replace them with relevant current references. Any changes to the reference list should be mentioned in the rebuttal letter that accompanies your revised manuscript. If you need to cite a retracted article, indicate the article’s retracted status in the References list and also include a citation and full reference for the retraction notice.

[Note: HTML markup is below. Please do not edit.]

Reviewers' comments:

Reviewer's Responses to Questions

**Comments to the Author**

1. Is the manuscript technically sound, and do the data support the conclusions?

The manuscript must describe a technically sound piece of scientific research with data that supports the conclusions. Experiments must have been conducted rigorously, with appropriate controls, replication, and sample sizes. The conclusions must be drawn appropriately based on the data presented.

Reviewer #1: Yes

Reviewer #2: Yes

Reviewer #3: Partly

**********

2. Has the statistical analysis been performed appropriately and rigorously?

Reviewer #1: Yes

Reviewer #2: Yes

Reviewer #3: No

**********

3. Have the authors made all data underlying the findings in their manuscript fully available?

The PLOS Data policy requires authors to make all data underlying the findings described in their manuscript fully available without restriction, with rare exception (please refer to the Data Availability Statement in the manuscript PDF file). The data should be provided as part of the manuscript or its supporting information, or deposited to a public repository. For example, in addition to summary statistics, the data points behind means, medians and variance measures should be available. If there are restrictions on publicly sharing data—e.g. participant privacy or use of data from a third party—those must be specified.

Reviewer #1: Yes

Reviewer #2: Yes

Reviewer #3: Yes

**********

4. Is the manuscript presented in an intelligible fashion and written in standard English?

PLOS ONE does not copyedit accepted manuscripts, so the language in submitted articles must be clear, correct, and unambiguous. Any typographical or grammatical errors should be corrected at revision, so please note any specific errors here.

Reviewer #1: Yes

Reviewer #2: Yes

Reviewer #3: Yes

**********

5. Review Comments to the Author

Please use the space provided to explain your answers to the questions above. You may also include additional comments for the author, including concerns about dual publication, research ethics, or publication ethics. (Please upload your review as an attachment if it exceeds 20,000 characters)

Reviewer #1: The manuscript “Accurate quantification of spliced and unspliced transcripts for single-cell RNA sequencing with tidesurf” by Schleicher et al. describes the implementation of a new tool, tidesurf, for RNA velocity inference. The key advance of this tool is its ability to infer RNA velocity measures from both 3′- and 5′-based scRNA-seq protocols. The authors demonstrate clear advantages of tidesurf over velocyto, which was not designed to handle 5′-based scRNA-seq protocols but has nevertheless been used for this purpose, often leading to misleading results. They present a strong and defensible case for the superiority of tidesurf in analyzing 5′-based scRNA-seq data by identifying the underlying issues that affect velocyto’s performance.

The authors also compare tidesurf with alevin-fry and STARsolo, showing high concordance between these tools for 5′-based scRNA-seq protocols and demonstrating that all tools perform similarly on 3′-based scRNA-seq. The performance benchmarks provided in the supplementary materials are additionally helpful for understanding how these tools compare depending on what resources an investigator may have at their disposal.

One area where differences remain between tidesurf and alevin-fry is in datasets derived from human donors. The authors address this, noting: “The human genome contains more repetitive sequences than the murine genome [21], which could lead to differences in the (pseudo-) alignments of Cell Ranger and alevin-fry, especially for intronic sequences. Furthermore, the total unspliced counts were considerably lower on the two 5′ datasets, rendering small absolute differences relatively more important. Finally, alevin-fry’s quantification of total counts differed slightly more from the Cell Ranger output than tidesurf’s (Fig. 3B–D).”

I wonder whether there is a dataset that could serve as a ground truth (even a simulated dataset) to more directly evaluate the performance of these tools and clarify the sources of similarity or divergence between tidesurf and alevin-fry. One possible candidate is the dataset described in PMID: 38782298, which includes both long-read and short-read data generated from the same 10x Chromium samples. Although this dataset is not human, and the authors suggest the discrepancies may be human-specific, it could still provide informative insights for readers seeking to understand how these tools behave under conditions where transcript structures are more fully resolved.

Reviewer #2: 1. Is the manuscript technically sound, and do the data support the conclusions?

Yes. The study is technically solid and the comparisons across datasets and tools are well done. The data generally support the main conclusion that velocyto performs poorly on 5′ scRNA-seq data, while tidesurf and other tools give more consistent results. The conclusions are reasonable based on the presented evidence.

2. Has the statistical analysis been performed appropriately and rigorously?

The analyses used (correlations, distribution comparisons, and velocity/PAGA comparisons) are appropriate for this type of benchmarking study. However, the statistical evaluation is mostly descriptive, and a bit more formal testing would improve clarity, although it is not critical.

3. Have the authors made all data underlying the findings fully available?

Yes. The datasets are clearly referenced with GEO/ArrayExpress accessions, and processed data and tools are described well enough to allow reproduction. Data availability is adequate.

4. Is the manuscript presented in an intelligible fashion and written in standard English?

Yes. The manuscript is clearly written and easy to follow. Only minor language polishing is needed in a few dense methodological sections.

5. Review comments to the author

The manuscript is well written and addresses an important issue in scRNA-seq velocity analysis. The findings are convincing and supported by multiple datasets and methods. T

A few minor points to improve the manuscript:

•The conclusions about “incorrect” assignments in velocyto could be slightly softened, since there is no direct ground truth.

•A small summary table comparing tool settings would improve readability.

Generally, this is a solid and useful methodological paper with clear practical relevance.

Reviewer #3: The manuscript talks about an interesting aspect of identifying spliced and unspliced RNA transcripts but I am little confused as to why the authors have downplayed Velocyto which looks like the crux of the entire manuscript. I understand that Velocyto has not been upgraded to include the pitfalls of 5’ chemistry but what if they do that upgrade soon and make Velocyto as powerful as 10X genomics softwares.

It would have been nice if the authors have introduced Tidesurf as the new command line software which addresses the pitfalls of existing softwares such as Velocyto in a sentence or two but not make it the crux of the entire manuscript.

Can the existing manuscript be rearranged and rewritten so as to put the focus completely on the USPs of Tidesurf highlighting the real time importance rather than downgrading an existing software which was introduced in 2018-2019. I would say few sentences about Velocyto are in harsh language which is not desired for the readers.

If that modification (major revision) can be done than the manuscript will be in a good shape and can be considered for publication.

**********

6. PLOS authors have the option to publish the peer review history of their article (what does this mean?). If published, this will include your full peer review and any attached files.

If you choose “no”, your identity will remain anonymous but your review may still be made public.

**Do you want your identity to be public for this peer review?**  For information about this choice, including consent withdrawal, please see our Privacy Policy.

Reviewer #1: No

Reviewer #2: **Yes:** Addis Tekaw

Reviewer #3: **Yes:** Dr. Gauri Awasthi

**********

[NOTE: If reviewer comments were submitted as an attachment file, they will be attached to this email and accessible via the submission site. Please log into your account, locate the manuscript record, and check for the action link "View Attachments". If this link does not appear, there are no attachment files.]

To ensure your figures meet our technical requirements, please review our figure guidelines: https://journals.plos.org/plosone/s/figures

You may also use PLOS’s free figure tool, NAAS, to help you prepare publication quality figures: https://journals.plos.org/plosone/s/figures#loc-tools-for-figure-preparation.

NAAS will assess whether your figures meet our technical requirements by comparing each figure against our figure specifications.

Attachment Submitted filename: PLOS One tidesurf - review.docx

# Author comment: Author response to Decision Letter 1

**DOI:** 10.1371/journal.pone.0355867.r002

2 Jul 2026

see response to reviewers file for details

Attachment Submitted filename: Schleicher_2026_rebuttal.pdf

# Peer review: Decision Letter 1

**DOI:** 10.1371/journal.pone.0355867.r003

**Authors:** Andreas Neueder

27 Jul 2026

Accurate quantification of spliced and unspliced transcripts for single-cell RNA sequencing with tidesurf.

PONE-D-26-15640R1

Dear Dr. Claassen,

We’re pleased to inform you that your manuscript has been judged scientifically suitable for publication and will be formally accepted for publication once it meets all outstanding technical requirements.

Within one week, you’ll receive an e-mail detailing the required amendments. When these have been addressed, you’ll receive a formal acceptance letter and your manuscript will be scheduled for publication.

An invoice will be generated when your article is formally accepted. Please note, if your institution has a publishing partnership with PLOS and your article meets the relevant criteria, all or part of your publication costs will be covered. Please make sure your user information is up-to-date by logging into Editorial Manager at Editorial Manager® and clicking the ‘Update My Information' link at the top of the page. For questions related to billing, please contact billing support.

If your institution or institutions have a press office, please notify them about your upcoming paper to help maximize its impact. If they’ll be preparing press materials, please inform our press team as soon as possible -- no later than 48 hours after receiving the formal acceptance. Your manuscript will remain under strict press embargo until 2 pm Eastern Time on the date of publication. For more information, please contact onepress@plos.org.

Kind regards,

Andreas Neueder

Academic Editor

PLOS One

Additional Editor Comments (optional):

Reviewers' comments:

Reviewer's Responses to Questions

**Comments to the Author**

1. If the authors have adequately addressed your comments raised in a previous round of review and you feel that this manuscript is now acceptable for publication, you may indicate that here to bypass the “Comments to the Author” section, enter your conflict of interest statement in the “Confidential to Editor” section, and submit your "Accept" recommendation.

Reviewer #1: All comments have been addressed

Reviewer #2: All comments have been addressed

Reviewer #3: All comments have been addressed

**********

2. Is the manuscript technically sound, and do the data support the conclusions?

The manuscript must describe a technically sound piece of scientific research with data that supports the conclusions. Experiments must have been conducted rigorously, with appropriate controls, replication, and sample sizes. The conclusions must be drawn appropriately based on the data presented.

Reviewer #1: Yes

Reviewer #2: Yes

Reviewer #3: Yes

**********

3. Has the statistical analysis been performed appropriately and rigorously?

Reviewer #1: Yes

Reviewer #2: Yes

Reviewer #3: Yes

**********

4. Have the authors made all data underlying the findings in their manuscript fully available?

The PLOS Data policy requires authors to make all data underlying the findings described in their manuscript fully available without restriction, with rare exception (please refer to the Data Availability Statement in the manuscript PDF file). The data should be provided as part of the manuscript or its supporting information, or deposited to a public repository. For example, in addition to summary statistics, the data points behind means, medians and variance measures should be available. If there are restrictions on publicly sharing data—e.g. participant privacy or use of data from a third party—those must be specified.

Reviewer #1: Yes

Reviewer #2: Yes

Reviewer #3: Yes

**********

5. Is the manuscript presented in an intelligible fashion and written in standard English?

PLOS ONE does not copyedit accepted manuscripts, so the language in submitted articles must be clear, correct, and unambiguous. Any typographical or grammatical errors should be corrected at revision, so please note any specific errors here.

Reviewer #1: Yes

Reviewer #2: Yes

Reviewer #3: Yes

**********

6. Review Comments to the Author

Please use the space provided to explain your answers to the questions above. You may also include additional comments for the author, including concerns about dual publication, research ethics, or publication ethics. (Please upload your review as an attachment if it exceeds 20,000 characters)

Reviewer #1: The authors went beyond what was expected from me and actually bring to light interesting differences between long-read and short-read sequencing in their response. I think this manuscript contributes to the understanding of 5' sequencing and brings a new tool to this assessment.

Reviewer #2: The authors have addressed my comments satisfactorily. The manuscript has been improved by adding formal statistical tests, clarifying the interpretation of the results, and softening conclusions where no direct ground truth is available. The addition of summary tables for tool settings also improves readability and reproducibility. Overall, this is a well-executed and practically relevant benchmarking study that provides useful guidance for RNA velocity analysis of 10x Genomics 5′ scRNA-seq data. I have no further major concerns and recommend the manuscript for publication after these revisions.

Reviewer #3: All comments have been addressed satisfactorily by the authors in the revised version. Recommended for acceptance.

**********

7. PLOS authors have the option to publish the peer review history of their article (what does this mean?). If published, this will include your full peer review and any attached files.

If you choose “no”, your identity will remain anonymous but your review may still be made public.

**Do you want your identity to be public for this peer review?**  For information about this choice, including consent withdrawal, please see our Privacy Policy.

Reviewer #1: **Yes:** Philip J. Moos

Reviewer #2: No

Reviewer #3: **Yes:** Dr. Gauri Awasthi

**********

# Editor report: Acceptance letter

**DOI:** 10.1371/journal.pone.0355867.r004

**Authors:** Andreas Neueder

PONE-D-26-15640R1

PLOS One

Dear Dr. Claassen,

I'm pleased to inform you that your manuscript has been deemed suitable for publication in PLOS One. Congratulations! Your manuscript is now being handed over to our production team.

At this stage, our production department will prepare your paper for publication. This includes ensuring the following:

* All references, tables, and figures are properly cited

* All relevant supporting information is included in the manuscript submission,

* There are no issues that prevent the paper from being properly typeset

You will receive further instructions from the production team, including instructions on how to review your proof when it is ready. Please keep in mind that we are working through a large volume of accepted articles, so please give us a few days to review your paper and let you know the next and final steps.

Lastly, if your institution or institutions have a press office, please let them know about your upcoming paper now to help maximize its impact. If they'll be preparing press materials, please inform our press team within the next 48 hours. Your manuscript will remain under strict press embargo until 2 pm Eastern Time on the date of publication. For more information, please contact onepress@plos.org.

You will receive an invoice from PLOS for your publication fee after your manuscript has reached the completed accept phase. If you receive an email requesting payment before acceptance or for any other service, this may be a phishing scheme. Learn how to identify phishing emails and protect your accounts at https://explore.plos.org/phishing.

If we can help with anything else, please email us at customercare@plos.org.

Thank you for submitting your work to PLOS ONE and supporting open access.

Kind regards,

PLOS ONE Editorial Office Staff

on behalf of

Prof. Dr. Andreas Neueder

Academic Editor

PLOS One
